# From Amphibian Extracts to Focused Libraries: An Algorithm-Based Strategy to Uncover Potent Multi-Target Anti-Alzheimer Peptides

**DOI:** 10.3390/biom16101458

**Published:** 2026-10-07

**Authors:** Álvaro José Rietmann, Iván Sanchis, Roque Spinelli, Javier Alejandro López, Álvaro S. Siano

**Affiliations:** 1 Laboratorio de Péptidos Bioactivos, Departamento de Química Orgánica, Facultad de Bioquímica y Ciencias Biológicas, Universidad Nacional del Litoral, Santa Fe 3000, Argentina; arietmann@fbcb.unl.edu.ar (Á.J.R.); sanchisivan@fbcb.unl.edu.ar (I.S.); rspinelli@fbcb.unl.edu.ar (R.S.); 2 Consejo Nacional de Investigaciones Científicas y Técnicas (CONICET), Godoy Cruz 2290, Ciudad Autónoma de Buenos Aires (CABA) 1425, Argentina; jalopez@inali.unl.edu.ar; 3 Grupo de Estudios Sobre Anfibios, Laboratorio de Biodiversidad y Conservación de Tetrápodos, Instituto Nacional de Limnología (INALI: CONICET-UNL), Santa Fe 3000, Argentina

**Keywords:** Alzheimer’s disease, natural products, peptides, rational selection, cholinesterase inhibitors

## Abstract

Alzheimer’s disease (AD) is a multifactorial neurodegenerative disorder, driving the search for multitarget-directed ligands (MTDLs) able to modulate multiple pathological pathways. Since amphibians represent a rich source of bioactive peptides, skin extracts from *Scinax nasicus* and *Dendropsophus nanus*, classified by sex, developmental stage, and season, were evaluated against key AD-related mechanisms, including cholinesterase inhibition, antioxidant activity, and Fe^2+^ chelation. Subsequently, a rational discovery workflow was implemented, integrating extract-level chemical characterization and bioactivity screening with the sequence identification of the main components and a novel in silico algorithm-based prioritization. This strategy, formalized through the Multi-Target Therapeutic Potential Score (MTTP-Score), served as a rational filter to produce a focused synthetic library, significantly minimizing experimental time and cost. Peptides derived from *D. nanus* (Dn) displayed the most promising multitarget candidates. Dn-7 combined sub-micromolar dual cholinesterase inhibition (IC_50_ = 0.32 and 1.2 µM for AChE and BChE, respectively) with highly efficient Fe^2+^ chelation (EC_50_ = 39 µM), representing the highest MTDL activity reported for an amphibian peptide. Dn-18 emerged as a moderate butyrylcholinesterase inhibitor with strong metal chelating capacity. Overall, this study establishes a pipeline for the rational discovery of peptide-based MTDL scaffolds for AD.

## 1. Introduction

Alzheimer’s disease (AD) is a progressive and irreversible neurodegenerative disorder that currently affects over 50 million people worldwide, a figure projected to triple by 2050, representing one of the most urgent needs in modern medicine [1,2]. The high failure rate of new drug candidates is largely attributed to the multifactorial nature of AD pathology, which involves cholinergic depletion, oxidative stress, dyshomeostasis of metal ions (Fe^2+^, Cu^2+^), and the accumulation of amyloid-beta (Aβ) aggregates, among others [3].

Acetylcholine (ACh) is a vital neurotransmitter for learning and memory, and its levels are drastically reduced in AD brains due to the rapid hydrolysis mediated by two cholinesterases: acetylcholinesterase (AChE) and butyrylcholinesterase (BChE) [4]. Although early symptomatic treatments focused solely on AChE inhibition, accumulating evidence suggests that BChE plays a compensatory role as AD progresses. While AChE levels decline in the advancing disease, BChE activity remains stable or even increases by up to 120% in the hippocampus and cortex, effectively taking over the hydrolysis of ACh [5]. Dual inhibition of both enzymes is therefore now considered a superior strategy to restore cholinergic levels across all disease stages.

Beyond their hydrolytic function, these enzymes are intricately linked to the amyloid cascade, the second major pathological hallmark of AD. AChE possesses a peripheral anionic site (PAS) that interacts with the beta-amyloid peptide (Aβ), acting as a “chaperone” that accelerates Aβ aggregation into neurotoxic fibrils [6]. Consequently, compounds capable of binding to both the catalytic and peripheral sites of AChE can essentially act as dual-functional agents, by restoring ACh levels and delaying amyloid deposition.

The multifactorial nature of AD also includes oxidative stress and metal dyshomeostasis. The AD brain is under constant attack from reactive oxygen species (ROS), a condition that is further exacerbated by the accumulation of redox-active metals (e.g., Fe^2+^, Cu^2+^) within amyloid plaques [7]. These metals not only catalyze the production of hydroxyl radicals via the Fenton reaction but also promote the precipitation of Aβ [8]. Taken together, these interconnected pathological mechanisms have driven a paradigm shift in AD drug discovery from the “one-molecule, one-target” approach, towards the design of Multi-Target Directed Ligands (MTDLs)—single molecules capable of simultaneously modulating distinct pathological pathways [3].

Despite the urgent need for such MTDLs, the discovery of novel scaffolds remains challenging. In this sense, nature continues to be one of the main resources in drug discovery [9]. The skin of anuran amphibians constitutes a remarkable chemical reservoir, evolved over millions of years to protect these animals against predators and pathogens. Frogs and toads have specialized glands which, in response to stressful conditions, produce secretions rich in bioactive peptides, exhibiting potent antimicrobial, antiviral, anti-proliferative, anti-parasitic, and immune-modulating activities [10,11]. They have also recently shown neuroprotective, antioxidant, and enzyme-inhibitory properties [12,13]. Interestingly, however, the composition of amphibian skin secretions is not static; it varies significantly in response to intrinsic factors (sex, developmental stage) and extrinsic variables (seasonality, environmental stress) [14,15].

In spite of this potential, the transition from crude extract to lead compound remains a bottleneck in peptide discovery [16]. To address this, the integration of bioinformatics and rational design is essential. While tools for predicting antimicrobial activity are widespread, algorithms designed to prioritize peptides with multi-target anti-Alzheimer potential are scarce [17].

In this work, we present a rational strategy to discover novel bioactive peptides isolated from amphibian skin. We sampled frogs inhabiting a seasonal, humid, mesothermal fluvial region in Argentina, characterized by a warmer and rainier season from September to February, which conditions amphibian activity [18]. We assessed the influence of sex and seasonality on the skin bioactivity profile through a comparative screening of skin extracts from *Dendropsophus nanus*, *Dendropsophus sanborni* (ultimately excluded from downstream analysis due to its low bioactivity profile) and *Scinax nasicus*, obtained during the cold season of 2022, and a previously reported extract of *D. nanus* from the warm season [19]. After sequencing the most active samples—and given the size of the peptide libraries generated from these amphibian-derived sequences (>140 non-redundant sequences)—structural, thermodynamic, and in silico pharmacological descriptors were integrated into a composite ranking metric, the MTTP-Score, to prioritize six candidates for chemical synthesis. This score functions as a rational selection strategy rather than a validated structure–activity model, and its performance is assessed here primarily through its ability to enrich a small subset for multi-target potency. Selected candidates were then synthesized and evaluated in vitro to identify promising multi-target-directed ligands (MTDLs).

## 2. Materials and Methods

### 2.1. Sampling and Preparation of Skin Extracts

Specimens of *Dendropsophus nanus* (Dn), *Dendropsophus sanborni* (Ds) and *Scinax nasicus* (Sn) were collected during the cold season (May 2022) at San José del Rincón, Santa Fe, Argentina (31°35′39″ S, 60°34′22″ W). The capture and experimental protocols were under the supervision of members of the bioethical committee of the Facultad de Bioquímica y Ciencias Biológicas—Universidad Nacional del Litoral (Argentina). All the frogs used in this work were manipulated following the recommendations of the American Society of Ichthyologists & Herpetologists [20].

After sampling, specimens were euthanized by hypothermia following standard bioethical guidelines; dorsal skins were immediately removed and weighed. Prior to extraction, they were pooled after classification by sampling, species, sex and developmental stage, yielding one pooled homogenate per group. Solvent extraction was performed following the protocol described by Siano et al. [21]. Briefly, skins were minced and homogenized in a solution of ethanol:water (60:40 *v*/*v*), acidified to pH 3–4 with acetic acid (Anedra Research AG, Buenos Aires, Argentina) in order to inhibit endogenous protease activity and preserve peptide integrity. The homogenates were centrifuged twice at 10,000× *g* for 20 min at 4 °C. The supernatants were collected, lyophilized, and stored at −20 °C.

Additionally, a bioactive extract of *D. nanus* (males) obtained in the warm season (February 2017) from the same location and following the same extraction technique, previously reported by our group as a potent source of multitarget agents [19], was included as a reference benchmark (“Dn-2017”) to evaluate seasonal consistency.

### 2.2. Chemical Characterization of the Extracts

To assess the chemical complexity and evaluate qualitative variations associated with sex and seasonality, the extracts were analyzed by Reversed-Phase High-Performance Liquid Chromatography (RP-HPLC). Separations were carried out on an Atlantis C_18_ column (5 µm, 4.6 × 150 mm; Waters Corp., Wexford, Ireland) with a 5–80% ACN/H_2_O gradient (0.8 mL min^−1^, 33 min). Absorbance was monitored at 220/280 nm (Waters 2489; Waters Corp., Milford, MA, USA). Data acquisition and peak integration were performed using the Breeze 2 software. Protein content was quantified by the bicinchoninic acid (BCA) assay at 562 nm, using a bovine serum albumin (BSA) standard calibration curve in the concentration range from 0.5 to 5 mg mL^−1^.

### 2.3. Sequence Identification

The peptide sequence identification of the active extracts was achieved using liquid chromatography coupled to tandem mass spectrometry (LC-TIMS-TOF MS/MS). The samples were processed and analyzed at the Advanced Mass Spectrometry Facility (Florida International University, Miami, FL, USA).

### 2.4. Rational Selection: The MTTP-Score

A rational workflow was applied to select the most promising candidates for synthesis. First, based on the primary screening results, only the peptide pools from the most active extracts were selected for in silico analysis, while extracts with low or null activity were excluded. The resulting *de novo* sequences from each active extract were subjected to redundancy filtering using CD-HIT (Cluster Database at High Identity with Tolerance) with a 90% identity cutoff [22]. After that, the 3D structures of the top peptides from each non-redundant library were predicted using the EvolutionaryScale language model (ESMFold) [23]. This method was chosen to generate accurate conformational structures, avoiding secondary structure overrepresentation, common in short peptides.

Docking simulations were then performed using the HADDOCK 2.4 web server against both cholinesterases: *Electrophorus electricus* AChE (PDB ID: 1EEA; selected for its high active site conservation and lower promiscuity) and human BChE (PDB ID: 1P0I) [24]. Next, the best clusters were submitted to the HawkDock server for energy minimization and rescoring using the MM/GBSA (Molecular Mechanics/Generalized Born Surface Area) method, in order to provide a rigorous estimation of the binding free energy [25].

Multitarget and physicochemical properties were predicted using specific algorithms: AnOxPePred for antioxidant and chelating potential, AMPep for antimicrobial activity, BBPPred for blood–brain barrier permeability, and HLPpred-Fuse for hemolytic toxicity prediction [26,27,28,29].

Finally, a Multi-Target Therapeutic Potential Score (MTTP-Score) was developed to rank the candidates. Individual parameters from both docking simulations and physicochemical/biological properties predictions were normalized to a 0–1 scale using Min–Max scaling (when applicable), where the most favorable predicted value across the library for any given parameter was assigned a value of 1, and the least favorable, a value of 0. The final MTTP-Score was calculated using a weighted equation designed to prioritize Alzheimer’s-specific targets (cholinesterase inhibition; weight = 2) followed by complementary disease-modifying mechanisms (oxidative stress/metal chelation; weight = 1.25) and antimicrobial/membrane-penetrating properties (weight = 1), while penalizing toxicity:

$$\mathrm{MTTP}-\mathrm{Score}=\frac{\left[2\left({∆G}_{\mathrm{AChE}}+{∆G}_{\mathrm{BChE}}\right)+1.25(P_{\mathrm{AOx}}+P_{\mathrm{Chel}}\right)+\left(P_{G+}+P_{G-}+P_{\mathrm{BBB}}\right)-P_{\mathrm{Hem}}]}{9.5}$$

where ΔG_n_ represents the normalized binding free energies for cholinesterases, and P_n_ denotes the predicted probability scores for the associated neuroprotective and membrane-interacting properties, including a subtraction penalty term for predicted hemolysis (P_Hem_) to filter out potentially toxic candidates. The denominator (9.5) corresponds to the “maximum attainable multi-target potential”, reached when every favorable descriptor is 1 and predicted hemolysis is 0. The complete evaluated sequence list and individual parameters are available in Supplementary Data S1.

The general physicochemical properties of the selected peptides, including molecular weight, theoretical net charge at pH 7.0, grand average of hydropathicity (GRAVY), aliphatic index, and instability index, were computed using the ProtParam tool available on the ExPASy proteomics server: https://web.expasy.org/protparam/ (accessed on 8 August 2026). The percentage of hydrophobic residues was calculated based on the primary sequence composition. Computational stability predictions were performed using specific machine-learning web servers: peptide stability in an intestine-like environment was assessed using the HLP server, while the in vivo half-life in human blood plasma was estimated using the PlifePred web server [30,31].

### 2.5. Peptide Synthesis

All the chemicals and reagents used in this section were purchased from Novabiochem (Merck KGaA, Darmstadt, Germany). Peptides were synthesized by Fmoc solid-phase peptide synthesis (SPPS) on a Rink amide MBHA resin to obtain C-terminal amidated peptides. Couplings were performed using N, N’-diisopropylcarbodiimide (DIC) and Oxyma Pure (ethyl cyano(hydroxyimino)acetate) to ensure efficient acylation and suppress racemization. Fmoc removal was achieved with 20% piperidine in DMF (*v*/*v*). Final cleavage from the resin and side-chain deprotection was achieved by a mixture of TFA/H_2_O/triisopropylsilane (TIS) (95: 2.5: 2.5) (*v*/*v*). After 3 h, the resin was filtered off, and the crude peptide was precipitated in dry cold diethyl ether, centrifuged, and washed several times with cold diethyl ether until the scavengers were removed. The product was then lyophilized twice. The synthetic peptides were purified by reverse phase HPLC using a C_18_ Jupiter Proteo Phenomenex semi-preparative column (10 μm, 90 Å, 250 × 10 mm; Phenomenex, Torrance, CA, USA) with a gradient of 5–80% acetonitrile in water containing 0.1% trifluoroacetic acid. Then, each peptide was analyzed by C_18_ RP-HPLC.

### 2.6. Bioactivity Assays

The biological activities of both crude extracts and synthetic peptides were evaluated using identical experimental protocols.

#### 2.6.1. Cholinesterase Inhibition Assay

The in vitro inhibitory activity against both cholinesterases was evaluated by following Ellman’s colorimetric assay [32]. The reaction was performed in a final volume of 100 μL with rhAChE (100 pM) or rhBChE (2 nM), respectively; 0.4 mM acetylthiocholine iodide (ATCI, Sigma-Aldrich, Gillingham, UK) or s-butyrylthiocholine iodide (BTCI, Sigma-Aldrich, Gillingham, UK); 0.33 mM 5,5-dithiobis (2-nitrobenzoic acid) (DTNB, Sigma-Aldrich, Gillingham, UK); and the sample at 1250–156 μg mL^−1^ (for extracts) or 400–25 μM (for synthetic peptides), in 0.1 M phosphate buffer (pH = 8.0). The enzyme and sample solutions were mixed and incubated for 15 min (room temperature) and the reaction was started by the addition of the substrate and DTNB solutions. Absorbance at 405 nm was measured after 5 min in a 96-well microplate reader. For both enzymes, galantamine (200 µM; Thermo Fisher Scientific, Waltham, MA, USA) was used as positive control.

#### 2.6.2. Antioxidant Activity

Antioxidant activity was determined by the 2,2′-diphenyl-1-picrylhydrazyl (DPPH) radical scavenging assay according to the method of Memarpoor-Yazdi adjusted with a final volume of 250 μL [33]. Fifty μl of decreasing concentrations of the samples (1000–62.5 μg mL^−1^ -for extracts- or 400–25 μM -for synthetic peptides-) were mixed with 150 μL methanol and 50 μL DPPH (0.15 mM in methanol; Sigma-Aldrich, St. Louis, MO, USA) and incubated for 30 min in the dark. The absorbance of the resulting solution was measured at 490 nm. Ascorbic acid (300 mM; Sigma-Aldrich, St. Louis, MO, USA) was used as positive control.

#### 2.6.3. Fe^2+^ Chelating Activity

Fe^2+^ chelating activity was measured following the method described by He et al. [34] with a final volume of 250 μL. Fifty μl of decreasing concentrations of the samples (1000–62.5 μg mL^−1^ for extracts or 400–25 μM for synthetic peptides) were premixed with 5 μL of ferrous sulfate solution (2 mM; Wiener Lab., Rosario, Argentina), and then 185 μL of distilled water was added and mixed vigorously. Then, 10 μL of ferrozine solution (5 mM; Wiener Lab., Rosario, Argentina) was added, mixed, and incubated at room temperature for 10 min. After incubation, the absorbance of the final solution was determined at 620 nm. EDTA (5 mM; Sigma-Aldrich, St. Louis, MO, USA) was used as positive control.

### 2.7. Data Analysis

All experiments were performed in technical triplicates. In all cases, biological activity was calculated as:

$$100-\left(\frac{A_{s}-A_{b}}{A_{0}-A_{b}}\right);$$

where A_s_ is the absorbance of the sample, A_b_ is the absorbance of the blank (negative control) and A_0_ is the absorbance of the control (no sample).

The creation of dose–response models, along with the IC_50_/EC_50_ calculations, was performed using IC50 Studio, an open-source R Shiny app recently developed by our group [35]. The data were fitted to standard sigmoidal models and the uncertainty of the calculated parameters was estimated using bootstrap methods with a 95% confidence interval.

## 3. Results

### 3.1. Chemical Characterization of the Extracts

Table 1 shows information about the collection of amphibian species in the 2022 sampling, classified according to sex and development stage, together with the corresponding extracts and the previously reported Dn-2017 [19].

The total protein content from each cutaneous extract was quantified by BCA assay to assess the peptidic richness of the samples (see Table 1). Since the extraction method involved the complete homogenization of the tissue in acidic ethanol, these values reflect the total pool of peptides stored in the granular and mucous glands, rather than just the fraction released upon stimulation. Protein recovery yields varied both across species and phenotypes. For *D. nanus* (2022), the male extracts displayed the highest protein content (58%), followed by females (50%) and juveniles (38%). Similarly, *D. sanborni* showed a comparable protein content (47%), while *S. nasicus* exhibited a lower protein recovery (36%). Previously, Dn-2017 (characterized under the same extraction and quantification protocol at the time of its collection) had presented a content of 48% [19], numerically similar to the 2022 Dn female extract.

**Figure 1:**
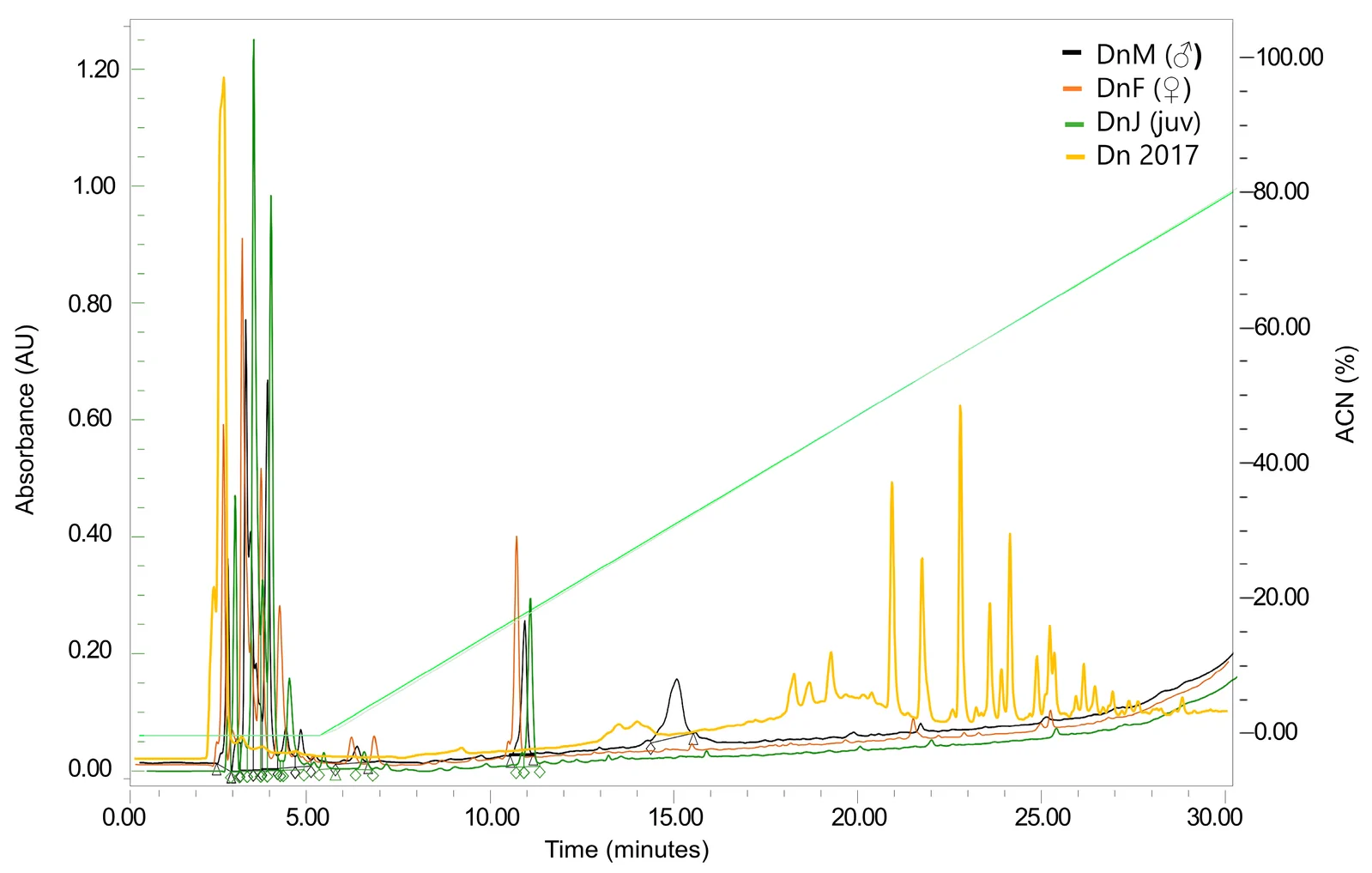
RP-HPLC profiles of *Dendropsophus nanus* skin extracts, monitored at 220 nm. The comparison highlights the chemical variability according to season, stage and sex. Elution conditions: C_18_ column, linear gradient 5–80% ACN/water with 0.1% TFA over 30 min.

Then, RP-HPLC profiles were analyzed (Figure 1, Figures S1 and S2). The chromatograms revealed distinct chemical fingerprints inter- and intra-species, despite using the same extraction method. Comparison between the previous extract (Dn-2017, warm season; Figure 1) and the samples here reported (Dn-2022, cold season; Figure 1) evidenced a shift in the elution pattern. Dn-2017 profile (analyzed under the same conditions at the time of its collection) was dominated by defined hydrophobic peaks (20–30 min), while Dn-2022 profiles showed a redistribution of components with new hydrophilic fractions (rt 3–15 min) and a simpler chromatogram overall. Within the same 2022 cold season cohort, intra-specific variation between adult male and female profiles was comparatively subtle: DnM and DnF showed similar elution patterns, with the exception of a distinctive peak at rt 15.07 min present exclusively in males. Finally, DnJ presented a remarkably similar fingerprint with comparable signal intensity.

The chromatographic profiles of Sn and Ds extracts, which showed different retention patterns characteristic of their species, are provided in the Supplementary Material (Figures S1 and S2).

### 3.2. Multi-Target Screening of the Extracts

The multi-target therapeutic potential of the crude extracts was evaluated against key AD pathological targets: cholinesterases (AChE, BChE), oxidative stress, and iron dyshomeostasis. The screening results are summarized in Table 2 and revealed a markedly heterogeneous activity landscape across species and phenotypes. These differences are presented as a general activity landscape for a subsequent bioactivity-guided selection, rather than as significant evidence of sex- or stage-specific effects.

At one extreme, *D. sanborni* (Ds) exhibited the poorest overall profile, with no detectable cholinesterase inhibition or chelating activity and a low antioxidant capacity (IC_50_ = 894 µg mL^−1^), suggesting that its extract lacks the structural diversity required for multi-target engagement under the tested conditions. At the other extreme, *D. nanus* (Dn) and *S. nasicus* (Sn) demonstrated broader and more potent activity profiles, with each species displaying distinctive strengths across the evaluated targets, as discussed in the sections below.

Regarding cholinesterase inhibition, Sn displayed the highest activity of the 2022 sampling (29% against AChE), and within Dn, females showed a slightly superior inhibition (25%) compared to males (22%) and juveniles (17%). However, no extracts produced a quantitative BChE inhibition. In comparison, the reference Dn-2017 confirmed the species’ capacity to produce potent neuroactive agents, exhibiting the highest cholinesterase inhibition of the entire set (IC_50_ = 309 µg mL^−1^ for AChE and 680 µg mL^−1^ for BChE) [19].

Beyond cholinesterase inhibition, the cold season 2022 extracts of Dn and Sn distinguished themselves through their non-enzymatic neuroprotective activities. DnM displayed the most relevant radical scavenging capacity among adult samples (EC_50_ = 494 µg mL^−1^). Notably, Dn-2017 presented no detectable antioxidant activity under the same conditions, further reinforcing the compositional differences between these seasonal samples noted above. Among all 2022 extracts, DnJ exhibited the highest radical scavenging capacity (EC_50_ = 252 µg mL^−1^), a result that contrasts sharply with its comparatively modest enzymatic inhibition.

Regarding Fe^2+^ chelating activity, DnM and Sn emerged as the most potent iron chelators (EC_50_ = 143 and 125 µg mL^−1^, respectively), followed by DnF and DnJ with similar but lower potencies (EC_50_ = 299 and 386 µg mL^−1^, respectively).

### 3.3. Rational Selection and Synthesis

Given the results of the bioactivity assays, the active extracts (Dn-2017, DnM, DnF, DnJ and Sn) were subjected to sequence identification to determine their peptidic composition, while Ds was omitted due to its negligible multi-target profile. The active fractions were analyzed via liquid chromatography-tandem mass spectrometry (LC-MS/MS). De novo sequencing of the resulting spectra yielded a primary library of over 140 unique peptide sequences across the studied extracts, which were subsequently subjected to virtual screening.

The transition from this primary library to a focused synthetic set required a rational prioritization framework. We developed the Multi-Target Therapeutic Potential Score (MTTP-Score), an integrated ranking system that combines thermodynamic binding estimates with a comprehensive pharmacological prediction profile, ensuring that selected candidates possess the delicate balance required to act as MTDLs.

**Figure 2:**
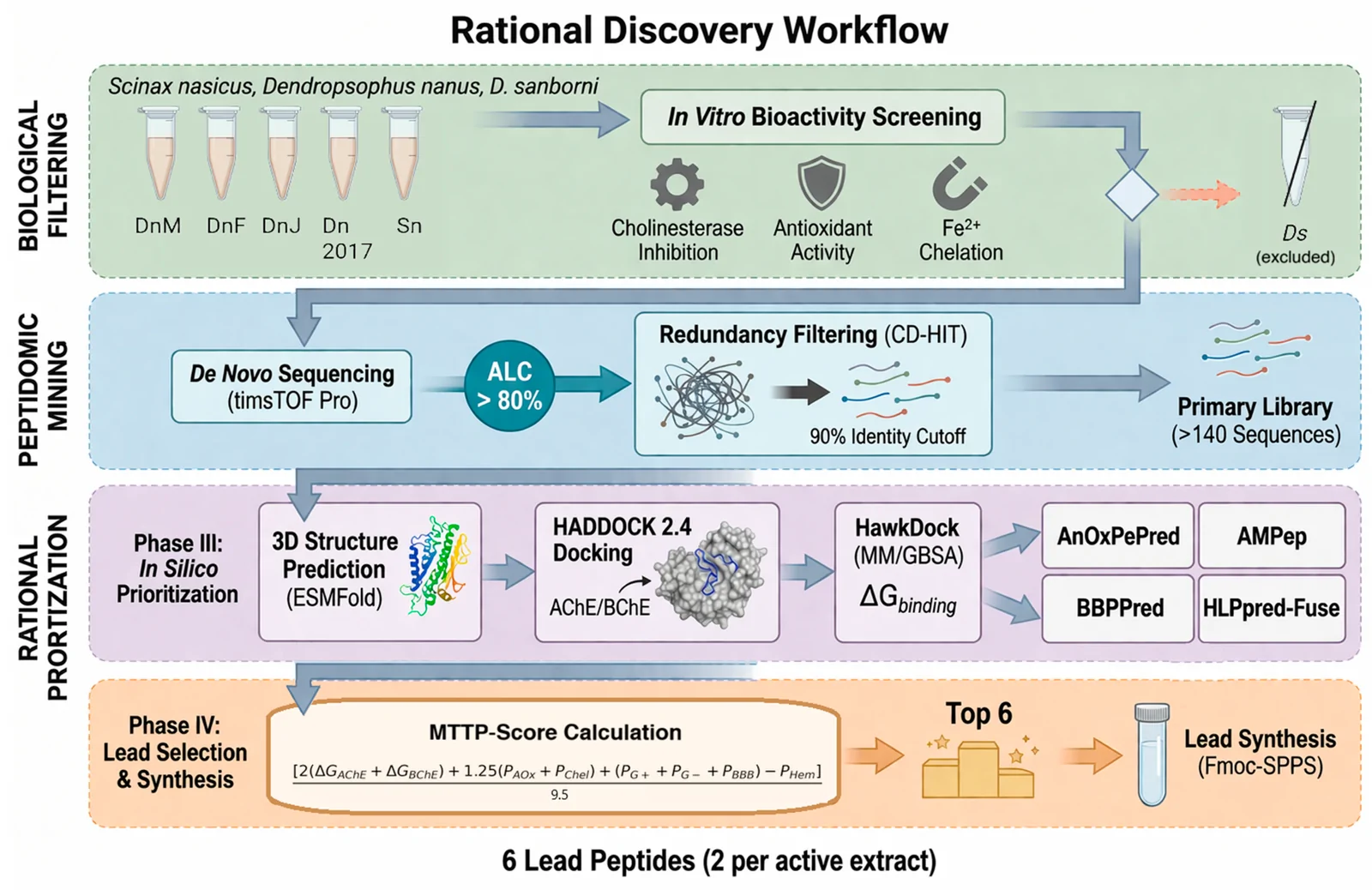
From extracts to leads. Schematic representation of the rational selection process, including extract-level biological filtering, peptidomic mining and the algorithm-based prioritization.

The rational screening workflow, schematically summarized in Figure 2, was implemented as follows. The primary library was first subjected to redundancy filtering using CD-HIT at a 90% identity cutoff [22], yielding three non-redundant peptide libraries classified by source extract: (i) Dn-2017, the previously characterized warm-season extract [19]; (ii) Dn-2022, stratified by sex and developmental stage (DnM, DnF, DnJ); and (iii) Sn, a single-sex pool. From each library, the top candidates were selected based on structural diversity, sequence length, net charge, and ALC score (≥80%), prioritizing sequences with features associated with target engagement. Their three-dimensional structures were predicted using ESMFold [36], a language model-based approach selected for its ability to generate accurate conformations for short peptides without the secondary structure overrepresentation common in homology-based methods. Candidates were then docked against eeAChE (PDB: 1EEA) and hBChE (PDB: 1P0I) using HADDOCK 2.4, and the resulting complexes were subjected to MM/GBSA rescoring via HawkDock to obtain rigorous binding free energy estimates [25]. In parallel, a multi-target pharmacological profile was mapped for each candidate using AnOxPePred (antioxidant and chelating potential), AMPep (antimicrobial activity), BBPPred (blood–brain barrier permeability), and HLPpred-Fuse (hemolytic toxicity) [26,27,28,29]. These structural, thermodynamic, and pharmacological descriptors were integrated into the MTTP-Score. To ensure phenotypic and structural diversity, the two top-ranked candidates from each non-redundant library were selected for chemical synthesis: six peptides in total.

The six selected sequences are shown in Table 3. Peptides were produced by Fmoc-SPPS on Rink amide MBHA resin, introducing a C-terminal amide in all cases. All synthetic candidates were purified by RP-HPLC and their molecular masses confirmed by MS, ensuring structural fidelity to the native sequence prior to biological evaluation.

### 3.4. Multi-Target Activity of Synthetic Peptides

The six synthesized candidates (Dn-7, Dn-18, DnF-26, DnF-33, Sn-2, and Sn-3) were evaluated in vitro across the same AD-relevant targets used in the extract screening. The biological profiles of the synthetic peptides are summarized in Table 4.

#### 3.4.1. Cholinesterase Inhibition

The transition from crude extracts to synthetic peptides produced a marked refinement of the cholinesterase inhibition profile. Several peptides derived from the Sn and Dn-2022 extracts (Sn-2, Sn-3, DnF-26, and DnF-33) maintained AChE inhibition levels comparable to those of the crude extracts (19–39% at 400 µM), but, more importantly, acquired moderate BChE inhibitory activity (13–29%), a feature absent in the crude extracts. This dual activity gain suggests that isolation and synthesis revealed active compounds that were either diluted or masked within the complex natural matrix. Nevertheless, despite this improvement, none of these peptides reached the potency threshold required for IC_50_ determination, underscoring their moderate biological relevance.

Remarkably, the highest activity emerged from peptides derived from the Dn-2017 extract, which had previously been identified as the most active crude sample (AChE IC_50_ = 309 µg mL^−1^ and BChE IC_50_ = 680 µg mL^−1^). Rational selection and synthesis of its lead peptides led to a pronounced increase in potency. Within this subset, Dn-18 displayed a marked preference for BChE: while its AChE inhibition was negligible (5% at 400 µM), it inhibited BChE by 41% at 400 µM (an approximately eightfold discrimination). The most striking outcome of the entire screening was the identification of Dn-7 as a high-affinity dual inhibitor. Dn-7 achieved sub-micromolar potency, with IC_50_ values of 0.32 ± 0.02 µM for AChE and 1.2 ± 0.1 µM for BChE (Table 4; 0.55 and 2.37 in µg mL^−1^, respectively), which constitutes a gain of approximately two to three orders of magnitude relative to the crude Dn-2017 extract. This strongly suggests that Dn-7 represents a principal bioactive component responsible for the neuroactive profile observed in the crude secretion.

It is noteworthy that the most potent cholinesterase inhibitor identified in this study, Dn-7, was also the top-ranked peptide according to the MTTP Score. Specifically, Dn-7 exhibited the most favorable predicted binding free energies (ΔG) for AChE and the second most favorable for BChE during the molecular modeling stage (Table 3).

#### 3.4.2. Antioxidant Activity

The synthetic peptide library was preliminarily evaluated for its radical scavenging capacity using the DPPH assay. The results indicated that most peptides possess mild-to-moderate scavenging potential (see Table 4). Peptides derived from the Sn and Dn-2022 series, specifically DnF-26, Sn-2 and Sn-3, displayed the highest activities within the group, scavenging 39% and 28% of free radicals, respectively, at the screening concentration (400 µM). This moderate efficacy is consistent with the antioxidant profile of the crude *S. nasicus* secretion (“Sn”), which exhibited an also moderate antioxidant EC_50_ of 408 µg mL^−1^ (Table 2), suggesting that these peptides are likely the main contributors to the overall antioxidant potential of crude extract.

Notably, Dn-7 and Dn-18 exhibited no detectable radical scavenging activity under these experimental conditions (Table 4). This result, however, not only mirrors the profile of their parent extract (Dn-2017), but is also consistent with the in silico predictions, in which these sequences received the lowest antioxidant scores of the synthesized library (Table 3).

#### 3.4.3. Fe^2+^ Chelating Activity

To assess this potential, the synthetic peptides were evaluated for their ability to chelate ferrous ions (Fe^2+^). In contrast to the modest antioxidant profile, the synthetic peptides displayed a more pronounced metal-chelating capacity. Most analogues (Sn-2, Sn-3, DnF-33, and DnF-26) exhibited a baseline chelating activity ranging from 32% to 42% at 400 µM (Table 4). Interestingly, the peptides derived from the Dn-2017 extract displayed a superior, dose-dependent efficacy. Dn-18 showed a high Fe^2+^ chelation capacity, reaching 50% activity at a low micromolar concentration (EC_50_ = 55.8 ± 0.2 µM). Remarkably, Dn-7 further improved this profile, demonstrating a two-fold higher potency, achieving the same level of Fe^2+^ chelation with an EC_50_ of 39 ± 1 µM, thus emerging as the most potent metal chelator of the peptide library.

Taken together, the bioactivity results of the synthetic peptides indicate that both Dn-18 and Dn-7 effectively target metal dyshomeostasis, a key upstream contributor to oxidative stress in AD, while also exhibiting complementary cholinesterase inhibition profiles. The convergence of potent Fe^2+^ chelation and dual enzymatic inhibition consolidates these peptides as the most promising MTDL scaffolds identified in this study, with the theoretical potential to simultaneously modulate interconnected enzymatic and non-enzymatic pathways involved in neurodegeneration.

Finally, regarding the relationship between predicted and actual in vitro activities, Spearman correlation between MTTP-Score and experimental potency across the six synthesized peptides showed negligible association with AChE inhibition but significant positive associations with BChE inhibition (ρ = 0.83, *p* = 0.042) and Fe^2+^-chelating activity (ρ = 0.93, *p* = 0.008). However, these values are reported only descriptively, since the main objective of this rational strategy is to serve as a bioactivity-guided enrichment tool.

### 3.5. In Silico Analysis of Structural, Physicochemical and Pharmacological Parameters of Dn-7 and Dn-18

The amino acid sequences and computed physicochemical characteristics of the top two candidates following the bioactivity assessment, Dn-7 and Dn-18, are summarized in Table 5. Dn-7 consists of a 17-amino-acid polypeptide chain with a net charge of +2 at pH 7 and a predominant proportion of hydrophobic residues (Hy/total aa = 58.8%). Based on the Kyte–Doolittle scale, Dn-7 possesses a Grand Average of Hydropathy (GRAVY) of 0.271 and an aliphatic index of 46.47, indicating a moderately hydrophobic and amphipathic profile.

**Figure 3:**
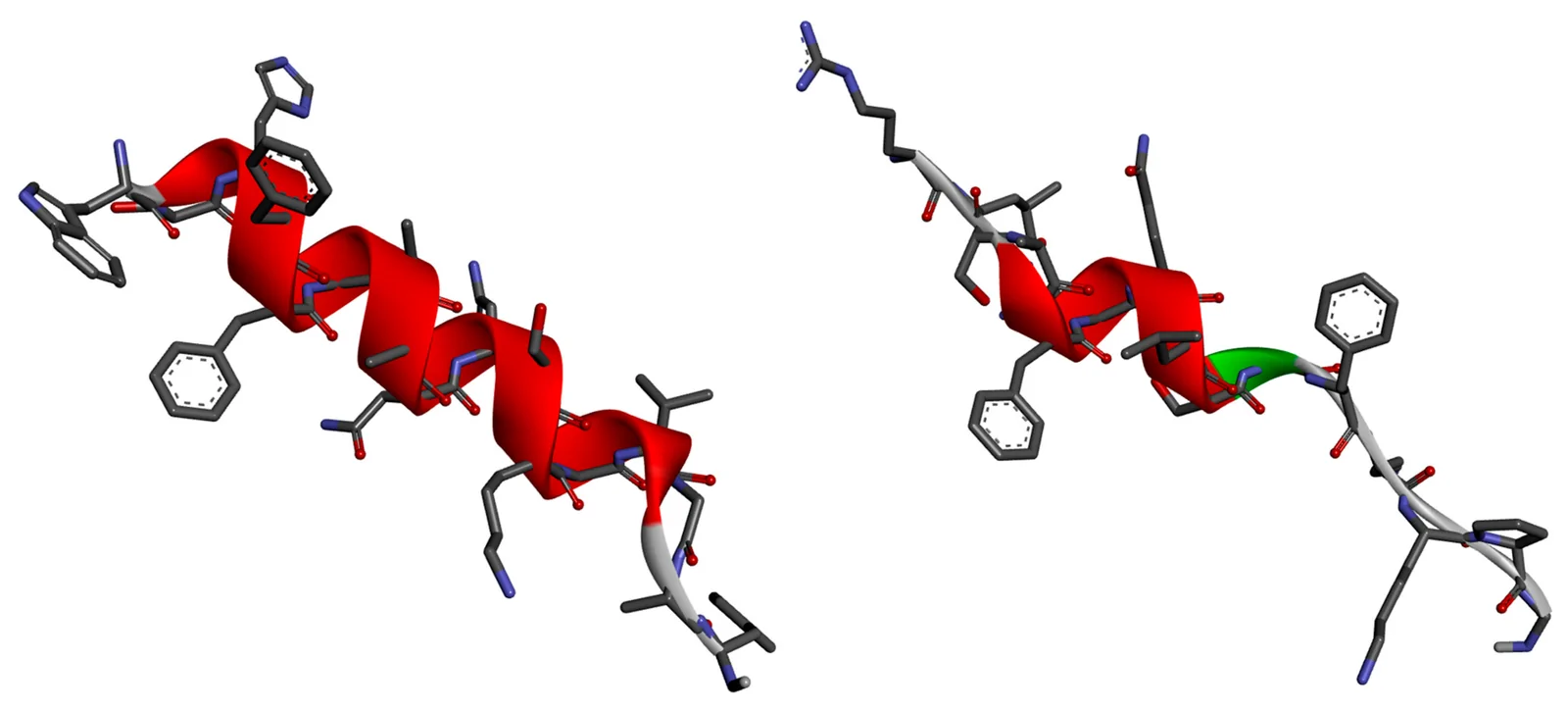
Peptides Dn-7 (**left**) and Dn-18 (**right**), predicted by Evolutionary Scale (ESMFold) server and visualized with Discovery Studio Software 2024 (BIOVIA). Colors indicate secondary structure: α-helix in red, turn in green, and coil in grey. Side chains are shown as sticks.

Conversely, Dn-18 is a 15-amino-acid peptide characterized by a highly cationic nature (net charge of +5 at pH 7) driven by the presence of arginine and lysine residues. It displays a distinctly hydrophilic profile, reflected in its negative GRAVY value (−0.620), a higher aliphatic index, and a lower hydrophobic amino acid content (33.3%). De novo 3D structural predictions using the ESMFold language model suggest that both sequences present a tendency to form ordered alpha-helical structures (Figure 3).

Beyond their fundamental physicochemical traits, the in silico pharmacological profiles generated during the MTTP-Score prioritization further contextualize their suitability as lead compounds. Both peptides exhibit low predicted hemolytic toxicity in silico (HLPpred-Fuse scores < 0.51), suggesting a favorable safety profile for cellular interactions, while also displaying promising computational blood–brain barrier (BBB) permeation predictions (see Table 3). HLP, PlifePred and ProtParam servers estimate good half-life and general stability scores for both Dn-7 and Dn-18 (Table 5), with a half-life of >1 s for intestine-like environments and instability indexes < 8. While these computational algorithms provide a rational, evidence-based prediction of the physiological behavior of Dn-7 and Dn-18, they will ultimately require in vitro and in vivo toxicological corroboration.

## 4. Discussion

### 4.1. Chemical Characterization of the Extracts

The chromatographic and protein-content profiles of the studied extracts revealed intra- and inter-species variability, consistent with the dynamic nature of amphibian defense systems. Overall, protein contents fall within the typical range reported for amphibian species from the region (18–50%), tending to be higher for Hylidae and Microhylidae families [19,21]. Within *D. nanus* (2022), the apparent gradient in protein percentages from adults to juveniles aligns with the previous literature suggesting a physiological correlation with gland maturation, where adults possess a larger reservoir of bioactive precursors than juveniles [37].

Regarding RP-HPLC, the most pronounced difference detected in this work was not between phenotypes but between seasons: the redistribution of components observed between the warm-season Dn-2017 profile (dominated by defined hydrophobic peaks) and the cold-season Dn-2022 profiles (in which new hydrophilic fractions appear and the overall chromatogram is simpler). Although a single-timepoint comparison, this pronounced qualitative difference is in line with the influence of extrinsic variables on the composition of amphibian skin secretions [14,15].

Within the same cold season cohort, intra-specific variation between adult males and females of *D. nanus* was comparatively subtle, the most distinctive feature being a peak eluting at 15.07 min present exclusively in males (DnM). This observation follows similar patterns already noted for other amphibians, including *Odontophrynus asper* (unpublished observations), *Rana splendida* and *Leptodactylus fallax* [38,39]. The chromatogram of DnJ was remarkably similar to that of the adults and of comparable signal intensity; however, considering the higher number of specimens sampled in this pool (*n* = 17) compared to adults (*n* = 3 each), this implies that the peptide content is likely lower in juveniles individually. This quantitative disparity would be consistent with a progressive maturation of the granular gland system, whose secretory capacity is known to increase with age and sexual development in anurans [37,40,41]. The apparently lower individual peptide content in juveniles may also reflect a distinct physiological priority at this developmental stage: juveniles undergo rapid somatic growth and inhabit shallow, UV-exposed waters, conditions associated with elevated endogenous ROS production. Consequently, rather than accumulating a diverse arsenal of defense peptides, the juvenile integument may prioritize a more focused antioxidant repertoire to protect developing tissues from oxidative damage [42].

### 4.2. Multi-Target Activity of the Extracts

The bioactivity landscape of the extracts is well contextualized within the broader literature on amphibian bioactive extracts. As the cold season 2022 extracts of Dn and Sn displayed modest MTDL potency, the reference extract Dn-2017 stood out as the most promising.

Regarding cholinesterase inhibition, the potency of Dn-2017 (IC_50_ = 309 μg mL^−1^ for AChE and 680 μg mL^−1^ for BChE) is comparable to crude extracts from *Pseudis platensis* and *Physalaemus santafecinus*, which are ranked among the most active reported. *P. platensis* showed IC_50_ values of 292 ± 2 (AChE) and 337 ± 8 (BChE), while *P. santafecinus* showed values of 348 ± 5 µg mL^−1^ (AChE) and 285 ± 7 µg mL^−1^ (BChE) [19]. The 2022-sampling results (<30% inhibition at 1250 µg mL^−1^) are consistent with many reports on extracts with moderate-to-low cholinesterase activity, often attributed to lower peptide richness or reduced abundance of hydrophobic, aromatic residues [12,13,19]. A recent example is the male extract of *O. asper*, which inhibited AChE and BChE by 40.2% and 44%, respectively, at 1250 µg mL^−1^ (unpublished data). This marked difference in cholinesterase inhibition properties between Dn-2017 and Dn-2022 samples is consistent with the distinct chromatographic profiles observed in Figure 1. The Dn-2017 extract was dominated by more hydrophobic components, a feature directly relevant to cholinesterase inhibition: the peripheral anionic site (PAS) of AChE is known to preferentially bind hydrophobic ligands through π-π stacking interactions [43] and the relative depletion of such compounds in the cold season 2022 profiles may be related to the reduced enzymatic activity observed.

As for the non-enzymatic neuroprotective activities, the cold season 2022 extracts of Dn and Sn proved to harbor radical scavenging and metal chelating agents. Amphibians possess a highly permeable, unkeratinized skin that exposes them to constant oxidative threats from both environmental and endogenous sources, driving the evolutionary development of efficient antioxidant defense systems, including a rich repertoire of antioxidant peptides (AOPs) [44,45]. In this context, the antioxidant capacity of DnM falls within the broad range reported for amphibian extracts: more potent than *P. santafecinus* (1238 µg mL^−1^) but less so than *Boana cordobae* (198 µg mL^−1^) or *Rhinella arenarum* (~7 µg mL^−1^) [12,19]. DnJ, in turn, exhibited the highest scavenging capacity of the 2022 set, which, as discussed previously, aligns with the physiological demands of the juvenile stage, where a less mature glandular system and increased UV exposure may favor the accumulation of antioxidant-active compounds over enzymatic inhibitors [42].

The Fe^2+^-chelating capacities determined for DnM and Sn rank among the highest reported for crude amphibian secretions, comparable to those of *O. asper* (EC_50_ = 107–156 µg mL^−1^; unpublished data) and *R. arenarum* (172 µg mL^−1^) [12], and establish the 2022 Dn and Sn extracts as interesting sources of metal-chelating bioactive peptides.

### 4.3. MTTP-Score as a Rational Selection Tool

The transition from complex crude extracts to isolated leads remains a critical bottleneck in natural product discovery. Classical abundance-based isolation approaches are poorly suited for multi-target discovery: a peptide present at low concentration in the extract may still constitute a high-affinity inhibitor, while a dominant component may be biologically inert. Moreover, while predictive tools for antimicrobial peptides are well established, relying on relatively simple physicochemical descriptors such as cationic charge and hydrophobic moment [46,47], algorithms specifically designed to rank candidates with multi-target anti-AD potential remain scarce. A successful anti-AD peptide must simultaneously interact with the active sites of cholinesterases, cross the blood–brain barrier, and offer antioxidant and metal-chelating properties [3]: a complexity that cannot be captured by any single existing predictor. To address this gap, the MTTP-Score was developed as a rational selection tool to aid the prioritization of a focused set of synthesizable peptides from a broader library of over 140 native sequences.

In contrast to many current sequence-based peptide screening workflows, in which candidate prioritization relies predominantly on pre-trained or dataset-derived models operating directly on peptide sequence and associated descriptors [48,49,50], the rational strategy based on the MTTP Score incorporates target-aware structural modeling, docking-based interaction estimates, and machine-learning-based predictions of chelating activity and other relevant properties. This hybrid design provides a broader and more mechanistically informed framework for prioritizing multifunctional peptides. At the same time, as emphasized in previous analyses of peptide bioinformatic tools, in silico predictions should be regarded primarily as an initial filtering strategy, since their outputs are strongly conditioned by the underlying algorithms and training datasets and may therefore differ across platforms; accordingly, such results are best interpreted as preliminary, hypothesis-generating evidence to guide candidate selection rather than as conclusive proof of biological activity [51].

Regarding the relationship between predicted and actual in vitro activities, two observations support the value of this approach as an enrichment strategy. First, the most potent cholinesterase inhibitor identified in this study, Dn-7, was also the top-ranked peptide of the entire evaluated set, exhibiting the most favorable predicted binding free energy for AChE and the second most favorable for BChE, after Dn-18. Second, there is a strong general agreement between extract-level bioactivity and the performance of the synthetic peptides: extracts classified as ‘moderate’ yielded analogues with intermediate efficacy, whereas the extract with the highest bioactivity across targets (Dn-2017) proved to be the definitive source of the highest-performing leads, Dn-7 and Dn-18. The Spearman correlations reported in Section 3.4 point in the same direction, showing positive associations between the MTTP-Score and both BChE inhibition and Fe^2+^-chelating activity, although with few synthesized peptides. These values are reported only descriptively.

### 4.4. Multi-Target Activity of Synthetic Peptides

#### 4.4.1. Cholinesterase Inhibition

The transition from crude extracts to synthetic peptides produced a marked refinement of the cholinesterase inhibition profile. Peptides derived from the Sn and Dn-2022 extracts (Sn-2, Sn-3, DnF-33 and DnF-26) maintained comparable AChE inhibition levels and acquired moderate BChE inhibitory activity compared to the crude extracts. Moderately active amphibian peptides against both cholinesterases have been previously reported. In one of the first reports for amphibians, Hp-1971 isolated from the skin of *Boana pulchella*, showed a 35% inhibition for BChE at a concentration of 400 µM but reaching only 20% inhibition for AChE at the same concentration. Its rationally designed analogue 7–21 surpassed 50% BChE inhibition at the same concentration, while maintaining low-to-moderate AChE activity (33% at the same concentration) [52]. Beyond amphibians, the 31-amino-acid peptide Snakin-Z isolated from *Ziziphus jujuba* fruit shows a dual inhibition at higher concentrations, with IC_50_ values of 580 µg mL^−1^ for AChE and 720 µg mL^−1^ for BChE [53], further illustrating moderate dual-activity among natural peptides.

Within the Dn-2017 subset, Dn-18 displayed a marked preference between the two enzymes, with negligible AChE inhibition and 41% inhibition of BChE at 400 µM. This preference is particularly relevant from a therapeutic standpoint, as BChE activity is known to increase during the progression of AD [54]. The most promising result was the dual-inhibition of Dn-7, whose potency places it among the most active peptide cholinesterase inhibitors described to date. It surpasses the potency of the recently reported, naturally occurring peptide isolated from *B. cordobae*, BcI-4, which inhibits AChE with an IC_50_ of 0.9 µM (for eeAChE) and 1.1 µM (for rhAChE), but lacks anti-BChE activity [55]. Dn-7 also exhibits stronger AChE affinity than the prominent W3 and W3W7 analogues derived from *B. pulchella*, which showed IC_50_ values of 0.99 µM and 1.70 µM, respectively [56]. Its dual inhibitory profile further distinguishes it. While W3W7 is recognized as one of the most potent peptidic BChE inhibitors (IC_50_ = 9.4 µM), Dn-7 demonstrates a superior capability against this target as well (IC_50_ = 1.2 µM), being approximately 7-fold more potent [43]. Most notably, when compared to the FDA-approved drug rivastigmine —whose reported IC_50_ under similar experimental conditions ranges between 0.39 and 32 µM for both cholinesterases—Dn-7 proves to be more effective in vitro [57]. Also, this concordance between predicted and observed bioactivity underscores the value of integrating complementary in silico descriptors derived from both peptide–enzyme interaction modeling and machine-learning-based predictions.

#### 4.4.2. Antioxidant Activity

Oxidative stress is central to the pathogenesis of AD, exacerbating neurodegeneration through lipid peroxidation, protein oxidation, and DNA damage, while also promoting Aβ aggregation and tau hyperphosphorylation [58]. An effective MTDL should therefore incorporate antioxidant properties capable of directly neutralizing ROS beyond its enzymatic inhibitory function [59].

In this case, the synthetic library displayed only mild-to-moderate scavenging potential, and the two leads (Dn-7 and Dn-18) showed no detectable activity. This last result, however, not only correlates to the profile of their parent extract (Dn-2017), and the in silico predictions for the MTTP-Score, but also with the lack of certain antioxidant residues. Effective radical scavenging in peptides strongly depends on the presence of electron-donating residues such as tyrosine, tryptophan, cysteine or histidine [60], absent in most sequences of this reported library. Naturally occurring peptides such as Temporin-MS1 and Temporin-TP1, which lack cysteine, tryptophan, or histidine residues, exhibit only mild radical scavenging capabilities (20–35%), likely driven by less active residues like methionine or phenylalanine [45,61]. Similarly, the cysteine-free amphibian peptide Andersonin-AOP9 displays significantly lower antioxidant potency compared to its cysteine-containing counterparts, confirming that the absence of these specific residues limits the scavenging potential [62].

This structural dependence, however, represents a starting point for rational optimization. A paradigmatic example is provided by the derivatives of the amphibian peptide Hp-1935: while the natural scaffold lacked significant antioxidant capacity, the strategic substitution of aliphatic residues with tryptophan yielded the analogues W3 and W3W7, which acquired potent DPPH scavenging activity, with W3W7 reaching efficacy levels comparable to ascorbic acid (EC_50_ = 27.58 ± 8.74 µM) [56]. A similar substitution strategy applied to Dn-7 and Dn-18 could selectively incorporate antioxidant capacity without compromising their established cholinesterase inhibition profile, further broadening their MTDL potential.

#### 4.4.3. Fe^2+^ Chelating Activity

Beyond radical scavenging, the restoration of metal homeostasis represents a complementary neuroprotective mechanism in AD. Redox-active transition metals, particularly iron (Fe^2+^) and copper (Cu^2+^), catalyze Fenton and Fenton-like reactions, leading to the generation of highly reactive hydroxyl radicals that amplify the oxidative stress cycle [63]. Also, these metal ions interact directly with β-amyloid peptides, accelerating their aggregation and stabilizing toxic oligomeric forms [64,65]. Therefore, MTDLs should offer a dual neuroprotective mechanism, neutralizing ROS generation while restoring metal homeostasis.

In contrast to their modest antioxidant profile, the synthetic peptides displayed a more pronounced metal-chelating capacity. The baseline activities observed for Sn-2, Sn-3, DnF-33 and DnF-26 are typical of short peptides capable of forming weak coordinate bonds with transition metals [66]. Peptides derived from Dn-2017, however, displayed a superior, dose-dependent efficacy, with Dn-7 emerging as the most potent metal chelator of the library (EC_50_ = 39 ± 1 µM).

Amphibians are known to tolerate heavy metals and persist in habitats contaminated with high concentrations of copper, zinc, arsenic, and cadmium, among other metals [67,68]. One of the possible contributions to this resistance is precisely the ability to chelate metals attributed to antioxidant peptides present in their skin. However, iron-chelating activity is a biological property rarely reported for amphibian skin peptides. BcI-4, derived from *B. cordobae*, was recently stated as the first frog-skin natural peptide with iron-chelating capability, reaching 50% chelation at 785 µM [55]. The potency displayed by Dn-7 (EC_50_ ≈ 40 μM) is notably superior, positioning it as a highly efficient chelator.

### 4.5. Structural Aspects of Dn-7 and Dn-18

The physicochemical and structural descriptors computed for Dn-7 and Dn-18 offer a partial rationalization of their distinct activity profiles. The moderately hydrophobic, amphipathic character of Dn-7 is consistent with the chromatographic behavior of its source extract, in which hydrophobic components dominated the late retention region of the highly active Dn-2017 profile, and is compatible with the preference of the AChE peripheral anionic site for hydrophobic ligands discussed above. Dn-18, in contrast, is more hydrophilic and highly cationic, a composition more associated with the coordination of metal ions than with engagement of the enzymatic gorge, which is in line with the activity profile determined experimentally. The tendency of both sequences to adopt ordered alpha-helical conformations is a common structural motif among bioactive amphibian peptides [69], likely providing the conformational stability required to effectively anchor into the complex enzymatic gorges of cholinesterases and establish the multifactorial interactions observed in bioactivity assays.

The in silico pharmacological descriptors described in Section 3.5 provide a preliminary and exclusively computational outlook. Reference thresholds for stable short peptides include a half-life of >1 s for intestine-like environments, ≈800 s for plasma, and an instability index <40 [70,71]. The values estimated for Dn-7 and Dn-18 meet all three criteria (Table 5), and their predicted hemolysis and blood–brain barrier permeability scores are also compatible with well-tolerated, brain-penetrant peptides. We emphasize, however, that these are predictions generated by machine-learning servers and were not verified experimentally in the present work.

## 5. Conclusions

The multifactorial nature of AD demands therapeutic strategies that go beyond single-target inhibition, yet the rational discovery of peptide-based MTDLs remains poorly explored. This work addresses that gap by presenting an integrated platform that connects the chemical diversity of amphibian skin products with a systematic, algorithm-guided prioritization framework. By combining extract-level bioactivity screening, high-resolution peptidomics, and a virtual screening algorithm based on the MTTP Score (which combines physicochemical metrics from molecular modeling with ML-based bioactivity predictions), we demonstrated that it is possible to progress from complex biological mixtures to a focused synthetic library in a fraction of the time and cost required by classical bioactivity-guided evaluation. Applied to a library exceeding 140 sequences, this rational strategy helped prioritize six peptides for synthesis, and the top-ranked candidates proved to be the most potent multi-target hits of the study. Broader validation across a larger synthesized set would be required before the MTTP-Score could be regarded as a generalizable potency predictor.

Peptides derived from *Dendropsophus nanus* (particularly those originating from the Dn-2017 extract) displayed the most relevant multitarget profiles. Dn-18 showed a moderate yet preferred BChE inhibition (a therapeutically relevant profile given the progressive increase in BChE activity during AD) combined with potent Fe^2+^ chelating capacity (EC_50_ = 56 µM). Dn-7, however, represents the most significant finding of this study. This naturally occurring 17-residue peptide (WSHFFAAANNSKAVGAF) simultaneously achieves sub-micromolar dual cholinesterase inhibition (IC_50_ = 0.32 and 1.2 µM for AChE and BChE, respectively) and highly efficient iron chelation (EC_50_ = 39 µM), a combination that, to the best of our knowledge, positions Dn-7 as one of the most potent amphibian MTDL hits reported to date for AD-relevant targets. These findings should nonetheless be interpreted within the scope of the present work. While the in vitro results and the in silico safety profiling provide a promising starting point, these preliminary results do not constitute definitive proof of biological safety or cellular efficacy. Consequently, further studies including anti-aggregation activity against Aβ, in vitro blood–brain barrier permeability, toxicity and serum stability assays represent natural next steps towards a deeper characterization of the potential of Dn-7 and Dn-18.

Ultimately, the amphibian skin, shaped by years of evolutionary pressure into a biochemical arsenal of remarkable complexity, proves to be a promising yet largely underexplored source of MTDL scaffolds. Dn-7 and Dn-18 now represent a starting point for subsequent studies focused on rational sequence optimization, detailed mechanistic characterization, and in-depth toxicological profiling, with the broader goal of translating these natural scaffolds into viable candidates for AD drug development.

## Figures and Tables

**Table 1:** Collection of amphibian specimens and their skin extracts.

Sampling	Location	Species	Extract	*n* (Specimens)	Protein %
Cold season 2022	San José del Rincón, Santa Fe, Argentina (31°35′39″ S, 60°34′22″ W)	*Dendropsophus nanus* (Dn-2022)	DnM (adult male)	3	58 ± 7
			DnF (adult female)	3	50 ± 4
			DnJ (juveniles)	17	38 ± 4
		*Dendropsophus sanborni* (Ds)	Ds (adult male)	3	47 ± 1
		*Scinax nasicus* (Sn)	Sn (adult female)	3	36 ± 5
Warm season 2017	San José del Rincón, Santa Fe, Argentina (31°36′16″ S, 60°36′28″ W)	*Dendropsophus nanus* (Dn-2017)	Dn-2017 ^1^ (adult male)	3	48 ± 2

^1^ previously reported by Spinelli et al. [19].

**Table 2:** Biological activity for the studied extracts across main AD targets.

	AChE		BChE		Antioxidant		Chelating	
	% ^1^	IC_50_ ^2^	% ^1^	IC_50_ ^2^	% ^1^	EC_50_ ^2^	% ^1^	EC_50_ ^2^
DnM	22%	-	-	-	61%	494 ± 42	82%	143 ± 8
DnF	25%	-	-	-	60%	577 ± 77	62%	299 ± 31
DnJ	17%	-	-	-	73%	252 ± 11	68%	386 ± 13
Ds	-	-	-	-	54%	894 ± 161	-	-
Sn	29%	-	-	-	78%	377 ± 15	87%	125 ± 22
Dn-2017 ^3^	75%	309 ± 9	66%	680 ± 10	-	-	N.A.	N.A.

^1^ at highest dose, 1250 µg mL^−1^; ^2^ µg mL^−1^; ^3^ previously reported by Spinelli et al. [19]. N.A.: not assayed.

**Table 3:** Algorithm-based multitarget prioritization of synthetic peptide candidates using the MTTP-Score.

Name	Sequence	ΔG (AChE) ^1^	ΔG (BChE) ^2^	Antioxidant (AnOxPePred)	Chelating (ChelPePred)	Gram- (AMPep) ^3^	Gram+ (AMPep) ^4^	BBB Penetration (BBPpred)	Hemolysis (HLPpred-Fuse)	MTTP-Score *
Dn-2017										
Dn-7	WSHFFAAANNSKAVGAF-NH_2_	1	0.939	0.305	0.210	0.907	0.913	0.548	0.165	0.708
Dn-18	RLSAFRQLSGFKKPG-NH_2_	0.501	1	0.394	0.215	0.948	1	0.582	0.514	0.608
Dn-2022										
DnF-33	VRLLSAPSGEAPMFV-NH_2_	0.385	0.705	0.428	0.232	0.664	0.709	0.607	0	0.523
DnF-26	VLLSAPSWADEFV-NH_2_	0.036	0.347	0.399	0.232	1	0.868	0.396	0	0.402
Sn-2022										
Sn-2	EGLAGPQGLEGR-NH_2_	0.391	0.361	0.471	0.214	0.932	0.641	0.105	0.004	0.425
Sn-3	KNGDANPDGEPGLL-NH_2_	0.046	0.447	0.393	0.256	0.158	0.054	0.493	0.002	0.263

^1^ Normalized ΔG values (AChE) using Min–Max scaling. The maximum value (lowest free energy) was −65.1 kcal/mol (Dn-7). ^2^ Normalized ΔG values (BChE) using Min–Max scaling. The maximum value (lowest free energy) was −74.33 kcal/mol (Dn-18). ^3^ Normalized predicted antimicrobial activity against Gram– bacteria using Min–Max scaling. The maximum value (lowest IC_50_) was 46.27 µM (DnF-26). ^4^ Normalized predicted antimicrobial activity against Gram+ bacteria using Min–Max scaling. The maximum value (lowest IC_50_) was 60.88 µM (Dn-18). * Only the two top-ranked sequences from each non-redundant library were selected for chemical synthesis.

**Table 4:** Biological activity for the synthesized peptides across main AD targets.

	AChE		BChE		Antioxidant		Chelating	
	% ^1^	IC_50_^2^	% ^1^	IC_50_^2^	% ^1^	EC_50_^2^	% ^1^	EC_50_^2^
Dn-7	100%	0.32 ± 0.02	100%	1.2 ± 0.1	-	-	92%	39 ± 1
Dn-18	5%	-	41%	-	-	-	68%	55.8 ± 0.2
DnF-33	21%	-	27%	-	-	-	37%	-
DnF-26	19%	-	29%	-	39%	-	32%	-
Sn-2	35%	-	18%	-	12%	-	42%	-
Sn-3	39%	-	13%	-	28%	-	32%	-

^1^ at highest dose, 400 µM; ^2^ µM.

**Table 5:** Physicochemical characterization of peptides Dn-7 and Dn-18.

Name	Sequence	MW (g/mol)	Net Charge (pH 7)	Hydrophobicity (%) ^1^	Aliphatic Index ^2^	GRAVY ^2^	Half-Life (Intestine-like; s) ^3^	Half-Life (Blood; s) ^4^	Instability Index ^2^
Dn-7	WSHFFAAANNSKAVGAF-NH_2_	1824	+2	58.8	46.47	0.271	1.131	779	−6.68
Dn-18	RLSAFRQLSGFKKPG-NH_2_	1691	+5	33.3	58.67	−0.62	1.595	951	7.13

^1^ Hy/total aa; ^2^ Calculated with Protparam tool: https://web.expasy.org/cgi-bin/protparam/protparam (accessed on 8 August 2026); ^3^ Calculated with HLP tool: http://crdd.osdd.net/cgibin/hlp/pep_both.pl (accessed on 8 August 2026); ^4^ Calculated with PlifePred tool: https://webs.iiitd.edu.in//raghava/plifepred/index.php (accessed on 8 August 2026).

## Data Availability

All data generated or analyzed during this study are included in this published article and/or its supplementary information file. Any other datasets are available from the corresponding author on reasonable request.

## Supplementary Materials

The following supporting information can be downloaded at https://www.mdpi.com/article/10.3390/biom16101458/s1, Supplementary Data S1: Complete evaluated sequence list for the MTTP-Score. Figure S1: RP-HPLC profile of *Dendropsophus sanborni* skin extract, monitored at 220 nm. Elution conditions: C_18_ column, linear gradient 5–80% ACN/water with 0.1% TFA over 30 min; Figure S2: RP-HPLC profile of *Scinax nasicus* skin extract, monitored at 220 nm. Elution conditions: C_18_ column, linear gradient 5–80% ACN/water with 0.1% TFA over 30 min; Figure S3: Antioxidant activity of the studied amphibian skin extracts vs. sample concentration, measured by percentage of DPPH scavenging. Points express the mean ± SD (n = 3); Figure S4: Metal chelating activity of the studied amphibian skin extracts vs. sample concentration, measured by percentage of Fe2+ chelation. Points express the mean ± SD (n = 3); Table S1: LC-TIMS-TOF MS analytical parameters and de novo sequencing scores for the selected peptides; Figure S5: MS1 for the peptide Dn-7 (*m*/*z* 912.9497; error < 5 ppm; sequence: WSHFFAAANNSKAVGAF). The main plot shows the mass-to-charge ratio (*m*/*z*) range highlighting the precursor ion and related fragments. The inset (top right) provides a detailed view of the isotopic distribution pattern; Figure S6: MS1 for the peptide Dn-18 (564.6554 *m*/*z*; error < 5 ppm; sequence: RLSAFRQLSGFKKPG). The main plot shows the mass-to-charge ratio (*m*/*z*) range highlighting the precursor ion and related fragments. The inset (top right) provides a detailed view of the isotopic distribution pattern; Figure S7: MS1 for the peptide DnF-33 (787.4198 *m*/*z*; error < 5 ppm; sequence: VRLLSAPSGEAPMFV). The main plot shows the mass-to-charge ratio (*m*/*z*) range highlighting the precursor ion and related fragments; Figure S8: MS1 for the peptide DnF-26 (717.3688 *m*/*z*; error < 5 ppm; sequence: VLLSAPSWADEFV). The main plot shows the mass-to-charge ratio (*m*/*z*) range highlighting the precursor ion and related fragments. The inset (top right) provides a detailed view of the isotopic distribution pattern; Figure S9: MS1 for the peptide Sn-2 (592.3037 *m*/*z*; error < 5 ppm; sequence: EGLAGPQGLEGR). The main plot shows the mass-to-charge ratio (*m*/*z*) range highlighting the precursor ion and related fragments. The inset (top right) provides a detailed view of the isotopic distribution pattern; Figure S10: MS1 for the peptide Sn-3 (698.8408 *m*/*z*; error < 5 ppm; sequence: KNGDANPDGEPGLL). The main plot shows the mass-to-charge ratio (*m*/*z*) range highlighting the precursor ion and related fragments. The inset (top right) provides a detailed view of the isotopic distribution pattern; Figure S11: AChE inhibitory activity of the synthesized peptides vs. sample concentration. Points express the mean ± SD (n = 3); Figure S12: AChE inhibitory activity of DnR-7 vs. sample concentration. Points express the mean ± SD (n = 3); Figure S13: BChE inhibitory activity of the synthesized peptides vs. sample concentration. Points express the mean ± SD (n = 3); Figure S14: BChE inhibitory activity of DnR-7 vs. sample concentration. Points express the mean ± SD (n = 3); Figure S15: Antioxidant activity of the synthesized peptides vs. sample concentration. Points express the mean ± SD (n = 3); Figure S16: Metal chelating activity of the synthesized peptides vs. sample concentration. Points express the mean ± SD (n = 3).
