# Supplementary material for: From Amphibian Extracts to Focused Libraries: An Algorithm-Based Strategy to Uncover Potent Multi-Target Anti-Alzheimer Peptides
Source: Biomolecules. 2026 Oct 7;16(10):1458. doi: 10.3390/biom16101458 (PMC13702688; doi:10.3390/biom16101458)
Supplement: Supplementary file 1 [file biomolecules-16-01458-s001.zip › Biomolecules - Supporting Information.pdf]

Supporting Information

Biomolecules

From Amphibian Extracts to Focused Libraries: An Algorithm-Based Strategy to Uncover Multi-Target Anti-Alzheimer Peptides

Álvaro Rietmann,[a,c] Iván Sanchis,[a,c] Roque Spinelli,[a,c] Javier Alejandro López,[b,c] and Álvaro Siano*[a,c]

[a] Álvaro Rietmann, Iván Sanchis, Roque Spinelli and Álvaro Siano
Laboratorio de Péptidos Bioactivos
Departamento de Química Orgánica, Facultad de Bioquímica y Ciencias Biológicas, Universidad Nacional del Litoral. Santa Fe, 3000, Argentina.
E-mail: asiano@fbcb.unl.edu.ar

[b] Javier Alejandro López
Grupo de estudios sobre Anfibios, Laboratorio de Biodiversidad y Conservación de Tetrápodos, Instituto Nacional de Limnología (INALI: CONICET-UNL)
Departamento de Ciencias Naturales, Facultad de Humanidades y Ciencias, Universidad Nacional del Litoral Santa Fe, 3000, Argentina.

[c] Álvaro Rietmann, Iván Sanchís, Roque Spinelli, Javier López and Álvaro Siano
Consejo Nacional de Investigaciones Científicas y Técnicas (CONICET), Argentina

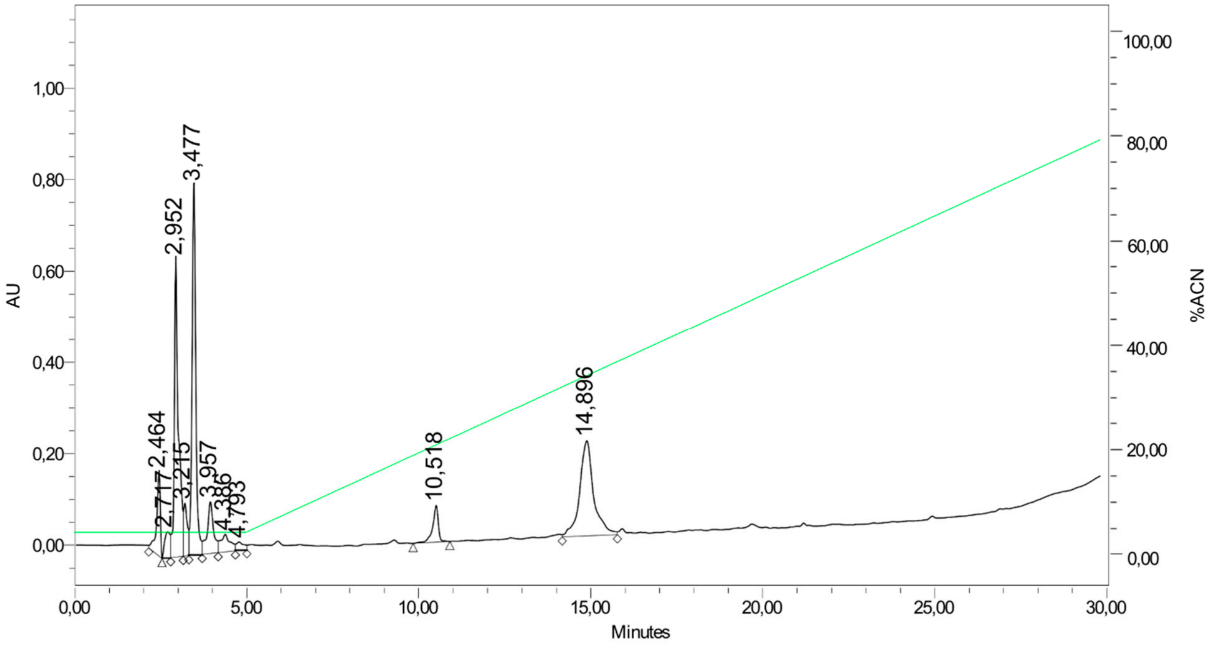

Figure S1. RP-HPLC profile of *Dendropsophus sanborni* skin extract, monitored at 220 nm. Elution conditions: C18 column, linear gradient 5–80% ACN/water with 0.1% TFA over 30 min.

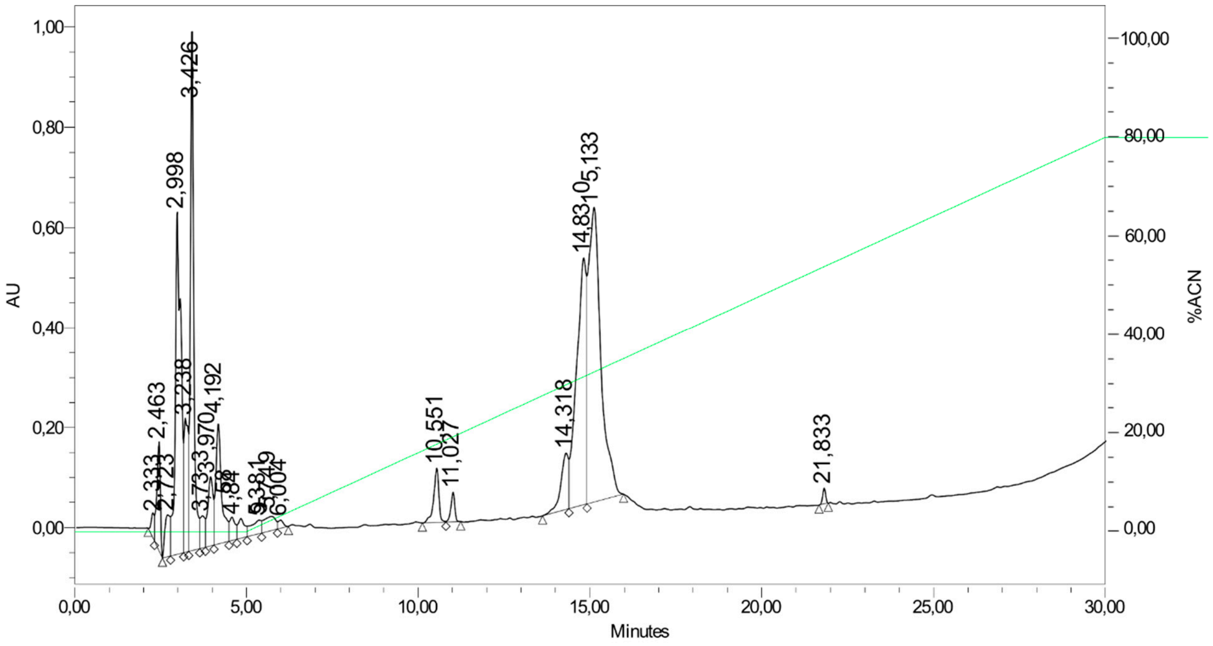

Figure S2. RP-HPLC profile of *Scinax nasicus* skin extract, monitored at 220 nm. Elution conditions: C18 column, linear gradient 5–80% ACN/water with 0.1% TFA over 30 min.

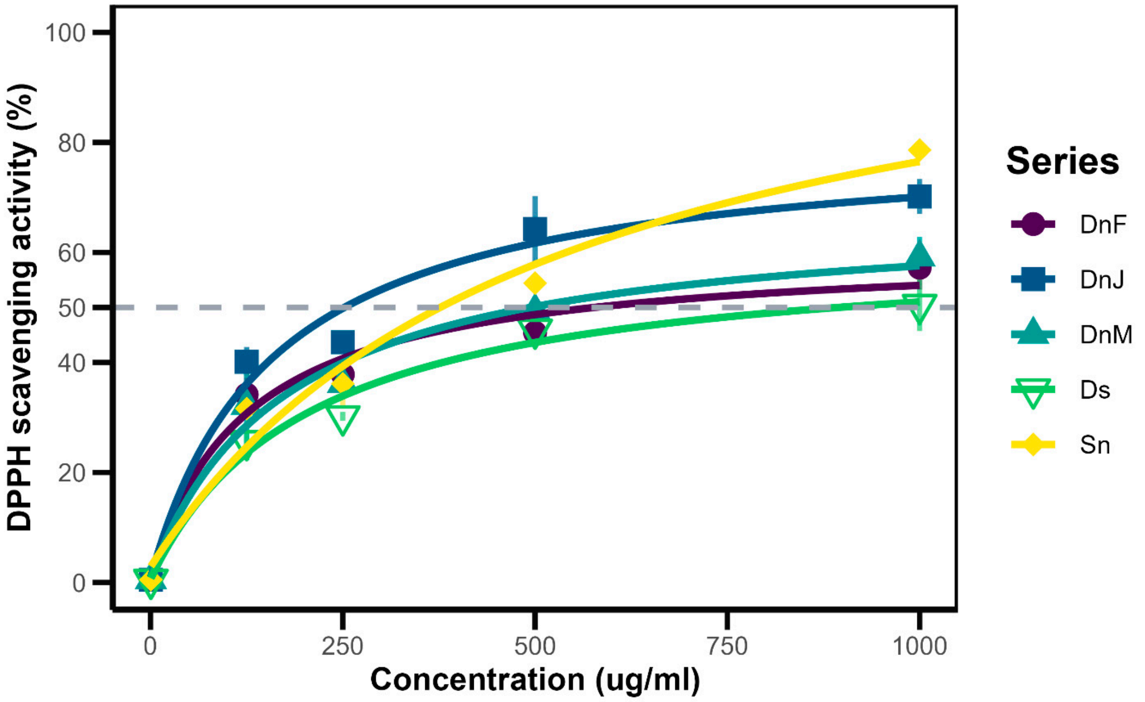

Figure S3. Antioxidant activity of the studied amphibian skin extracts vs. sample concentration, measured by percentage of DPPH scavenging. Points express the mean ± SD (n=3).

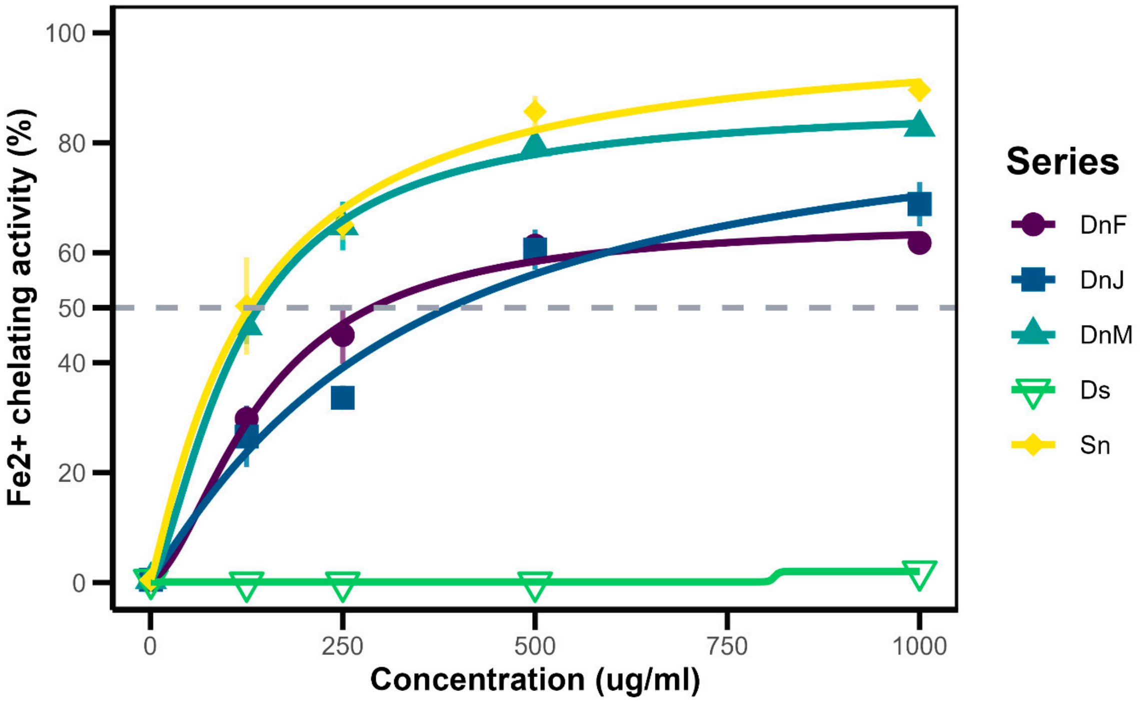

Figure S4. Metal chelating activity of the studied amphibian skin extracts vs. sample concentration, measured by percentage of Fe^{2+} chelation. Points express the mean ± SD (n=3).

Table S1. LC-TIMS-TOF MS analytical parameters and de novo sequencing scores for the selected peptides

	**Dn-7**	**Dn-18**	**DnF-33**	**DnF-26**	**Sn-2**	**Sn-3**
**Precursor *m/z* (charge)**[1]	912.9480 (2+)	564.6605 (3+)	787.4207 (2+)	717.3704 (2+)	592.3055 (2+)	698.8405 (2+)
**Theoretical monoisotopic mass (Da)**	1823.8743	1690.9631	1572.8333	1432.7239	1182.5994	1395.6631
**Mass error (ppm)**	+3.9	-2.1	-4.1	+1.6	-2.5	+2.3
**Retention time (min)**	29.32	20.72	22.82	21.93	15.81	17.97
**$1/K_0$ range**	1.2253-1.2519	0.8746-0.9022	1.0245-1.0517	0.9938-1.0211	0.8491-0.8769	0.9642-0.9916
**ALC / de novo score**	92	85	80	82	91	86

[1] Precursor *m/z*, mass error and scores as reported by the de novo sequencing software from the isotope-fitted precursor feature. *m/z* values annotated in the MS1 spectra of Figures S5–S10 may differ by up to ~2 ppm owing to peak picking and calibration. Mass error is calculated as (observed − theoretical)/theoretical.

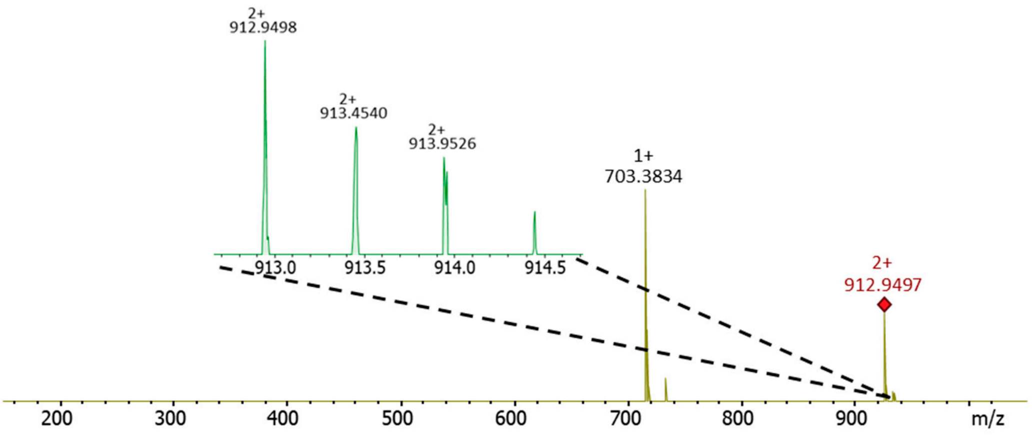

Figure S5. MS1 for the peptide Dn-7 (m/z 912.9497; error < 5 ppm; sequence: WSHFFAAANNSKAVGAF). The main plot shows the mass-to-charge ratio (m/z) range highlighting the precursor ion and related fragments. The inset (top right) provides a detailed view of the isotopic distribution pattern.

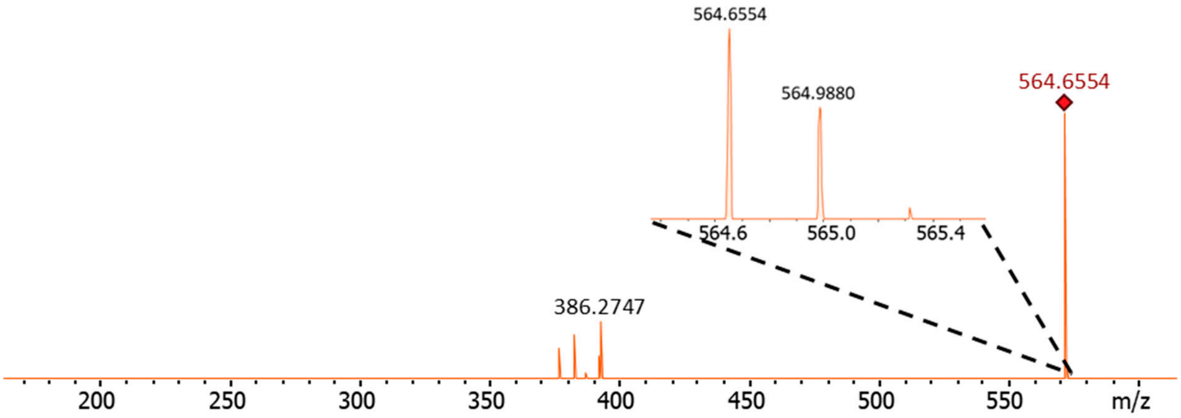

Figure S6. MS1 for the peptide Dn-18 (564.6554 m/z; error < 5 ppm; sequence: RLSAFRQLSGFKKPG). The main plot shows the mass-to-charge ratio (m/z) range highlighting the precursor ion and related fragments. The inset (top right) provides a detailed view of the isotopic distribution pattern.

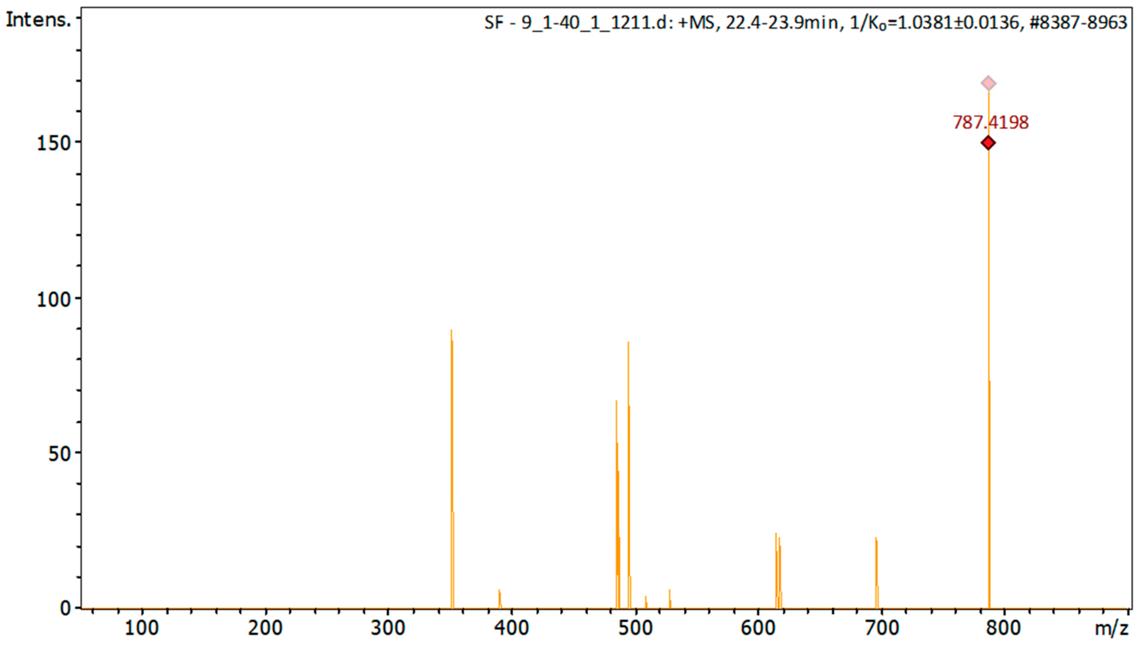

Figure S7. MS1 for the peptide DnF-33 (787.4198 m/z; error < 5 ppm; sequence: VRLLSAPSGEAPMFV). The main plot shows the mass-to-charge ratio (m/z) range highlighting the precursor ion and related fragments.

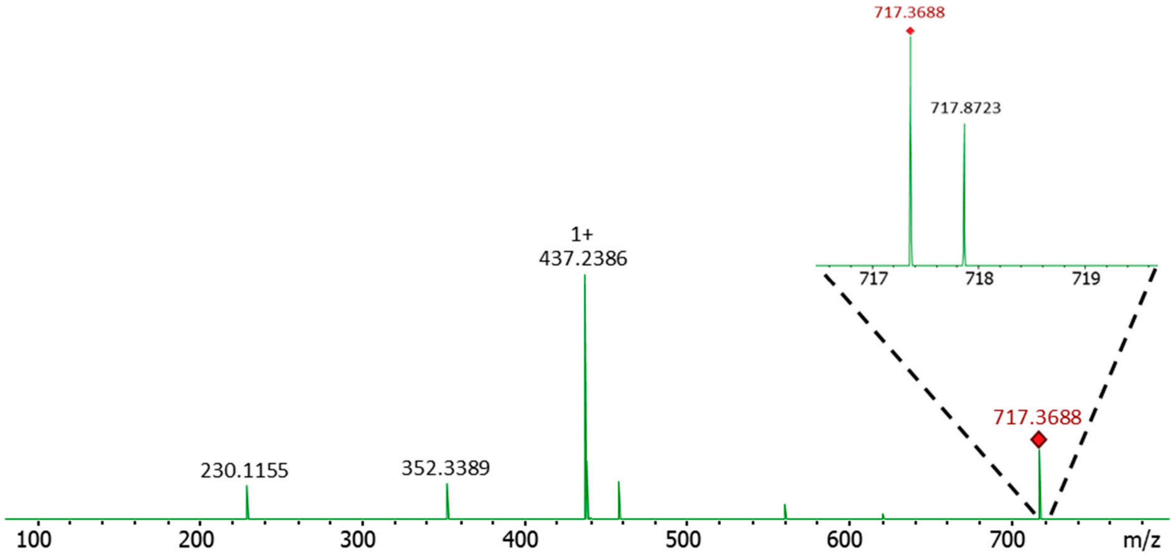

Figure S8. MS1 for the peptide DnF-26 (717.3688 m/z; error < 5 ppm; sequence: VLLSAPSWADEFV). The main plot shows the mass-to-charge ratio (m/z) range highlighting the precursor ion and related fragments. The inset (top right) provides a detailed view of the isotopic distribution pattern.

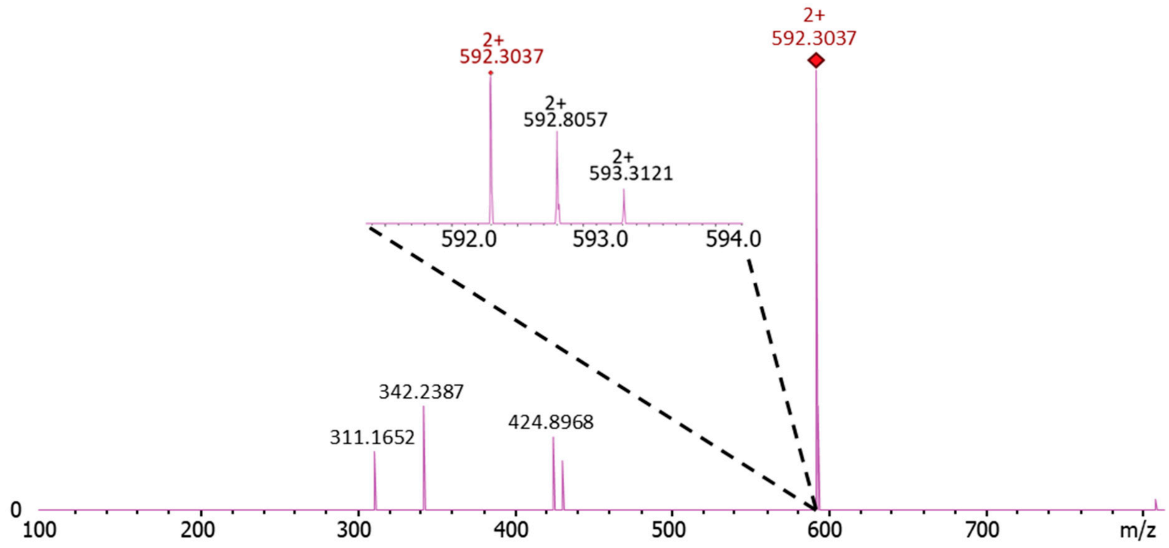

Figure S9. MS1 for the peptide Sn-2 (592.3037 m/z; error < 5 ppm; sequence: EGLAGPQGLEGR). The main plot shows the mass-to-charge ratio (m/z) range highlighting the precursor ion and related fragments. The inset (top right) provides a detailed view of the isotopic distribution pattern.

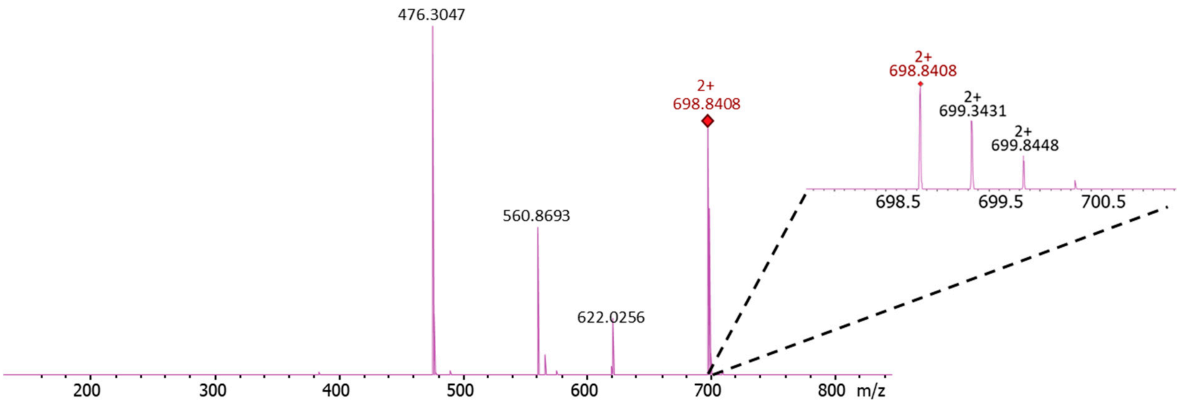

Figure S10. MS1 for the peptide Sn-3 (698.8408 m/z; error < 5 ppm; sequence: KNGDANPDGEPGLL). The main plot shows the mass-to-charge ratio (m/z) range highlighting the precursor ion and related fragments. The inset (top right) provides a detailed view of the isotopic distribution pattern.

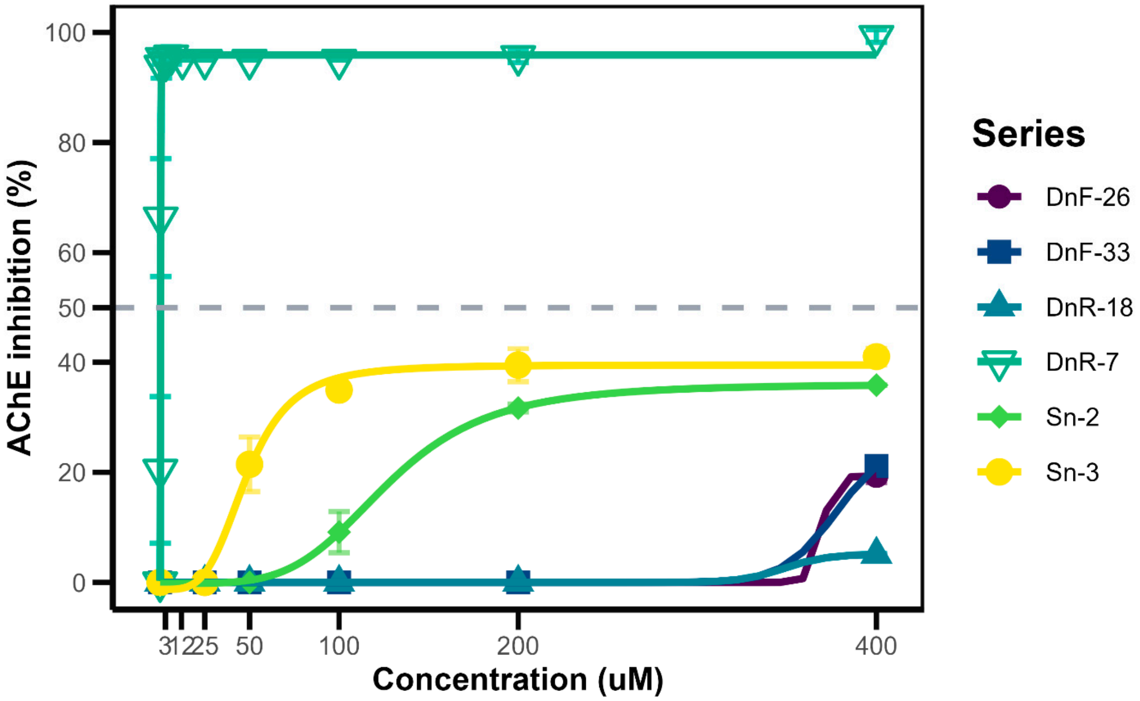

Figure S11. AChE inhibitory activity of the synthesized peptides vs. sample concentration. Points express the mean ± SD (n=3).

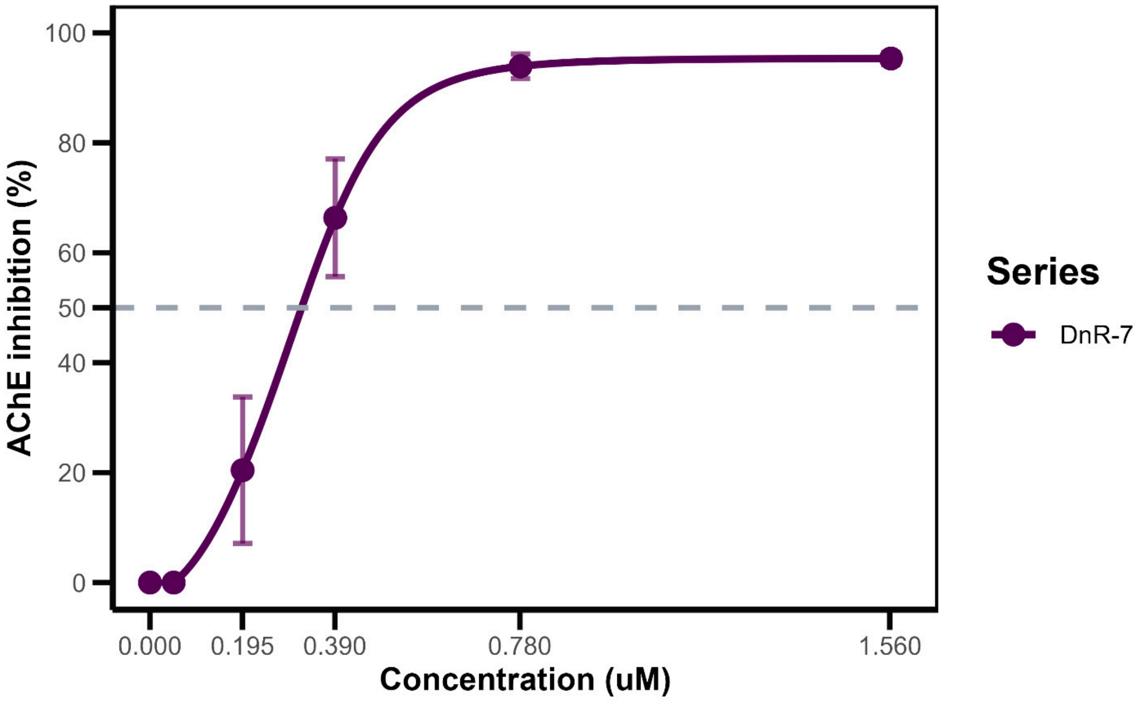

Figure S12. AChE inhibitory activity of DnR-7 vs. sample concentration. Points express the mean ± SD (n=3).

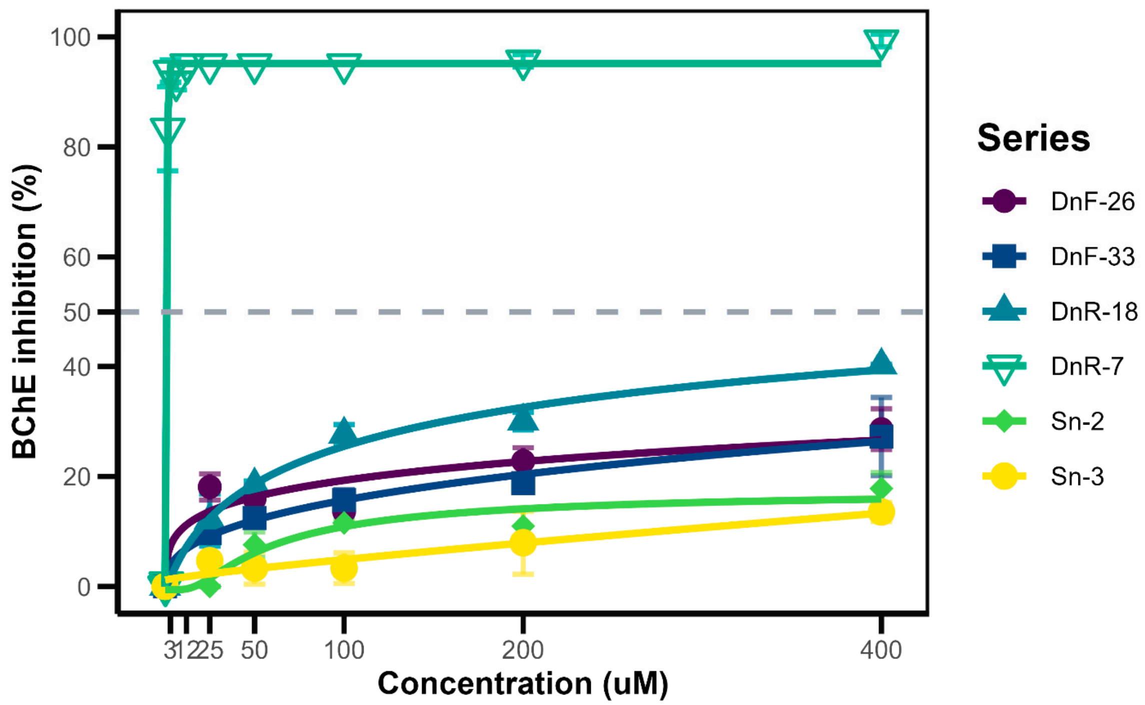

Figure S13. BChE inhibitory activity of the synthesized peptides vs. sample concentration. Points express the mean ± SD (n=3).

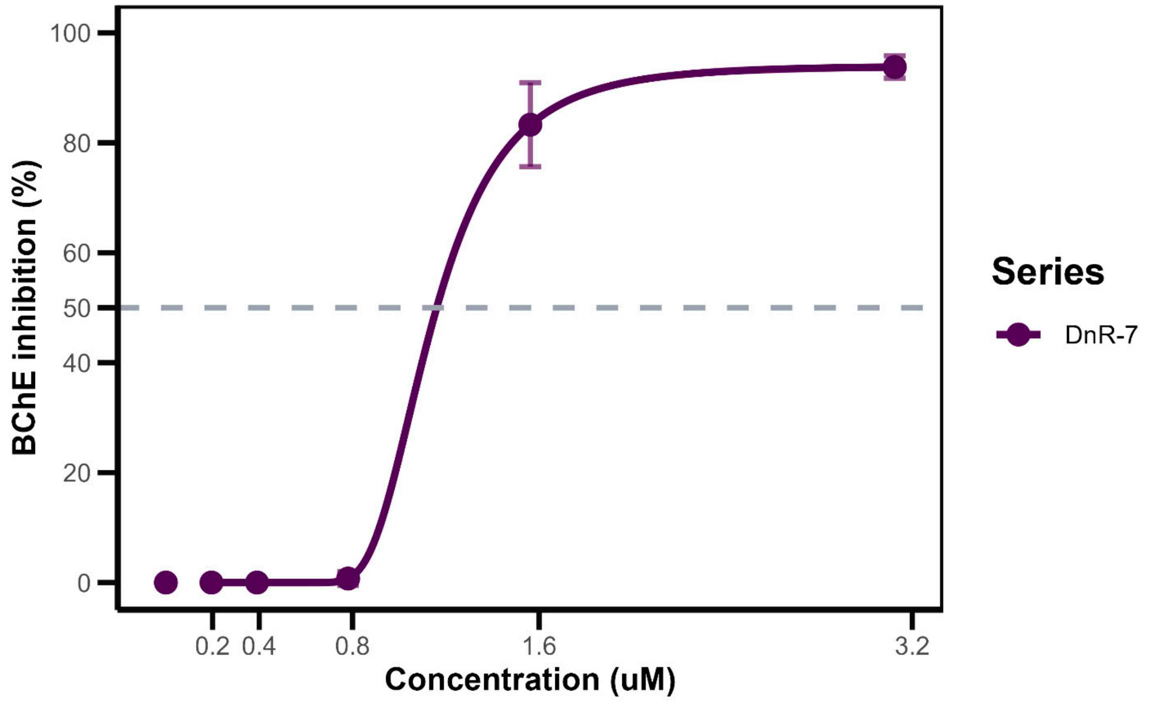

Figure S14. BChE inhibitory activity of DnR-7 vs. sample concentration. Points express the mean ± SD (n=3).

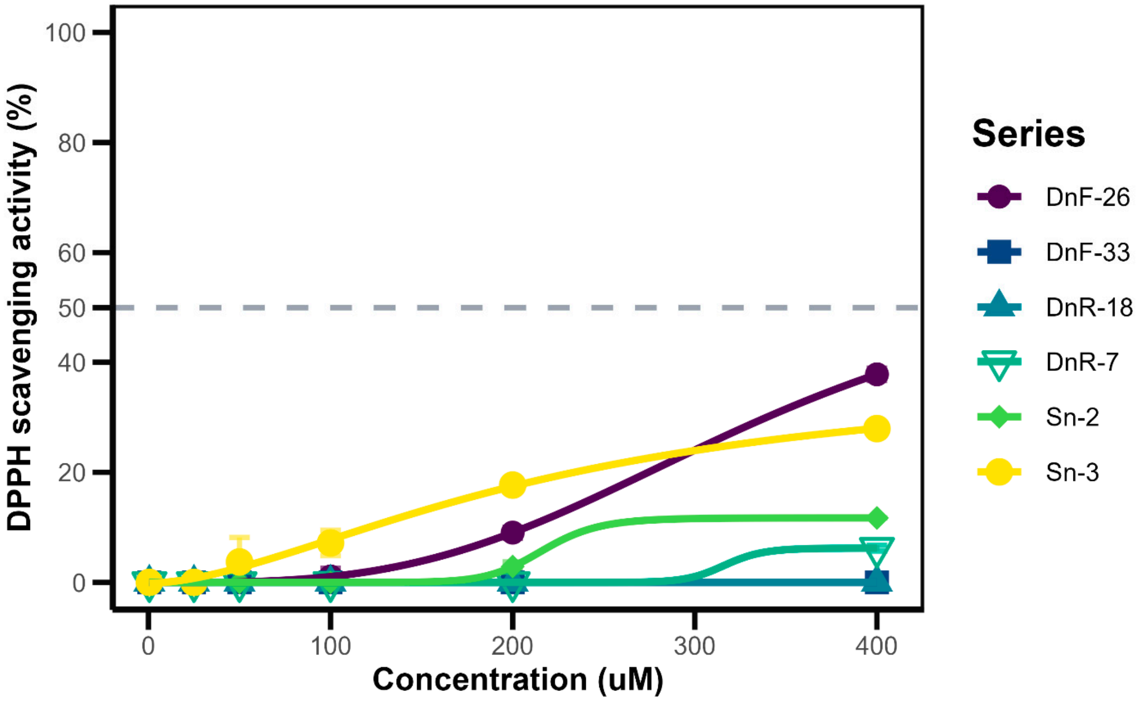

Figure S15. Antioxidant activity of the synthesized peptides vs. sample concentration. Points express the mean ± SD (n=3).

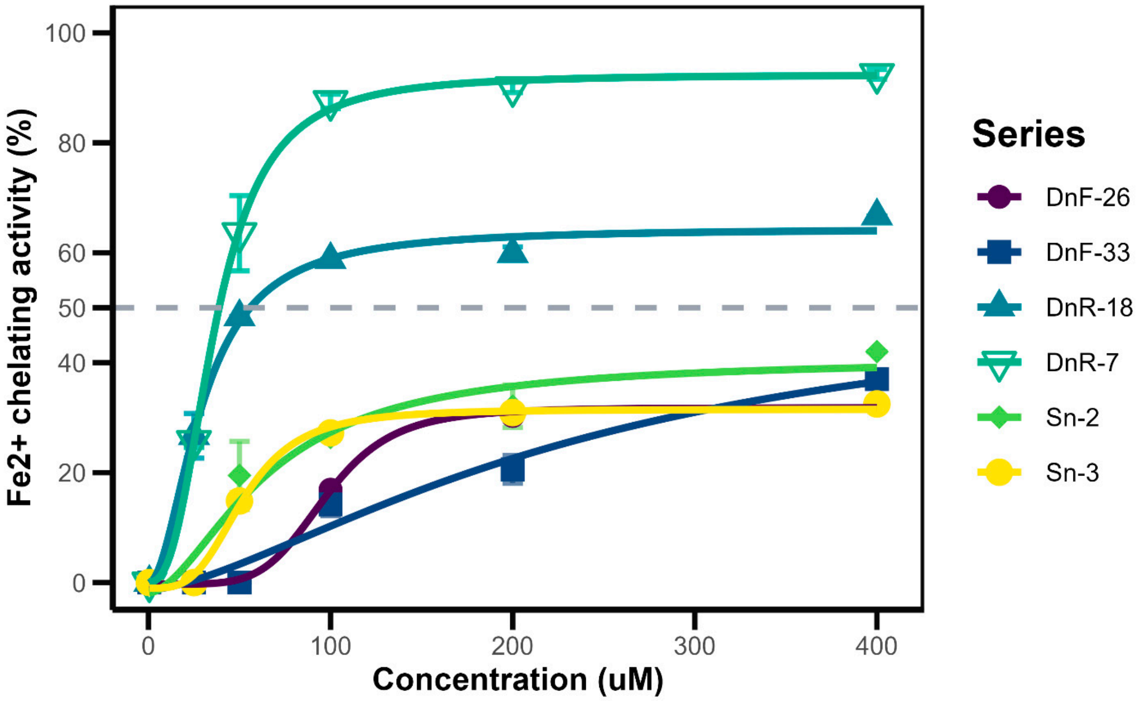

Figure S16. Metal chelating activity of the synthesized peptides vs. sample concentration. Points express the mean ± SD (n=3).
